# 1,2,3-Triazoles as leaving groups: S_N_Ar reactions of 2,6-bistriazolylpurines with O- and C-nucleophiles

**DOI:** 10.3762/bjoc.17.37

**Published:** 2021-02-11

**Authors:** Dace Cīrule, Irina Novosjolova, Ērika Bizdēna, Māris Turks

**Affiliations:** 1 Faculty of Materials Science and Applied Chemistry, Riga Technical University, P. Valdena Str. 3, LV-1048 Riga, Latvia

**Keywords:** 2,6-bistriazolyl purines, nucleophilic aromatic substitution, purine nucleosides, triazoles

## Abstract

A new approach was designed for the synthesis of C6-substituted 2-triazolylpurine derivatives. A series of substituted products was obtained in S_N_Ar reactions between 2,6-bistriazolylpurine derivatives and O- and C-nucleophiles under mild conditions. The products were isolated in yields up to 87%. The developed C–O and C–C bond forming reactions clearly show the ability of the 1,2,3-triazolyl ring at the C6 position of purine to act as leaving group.

## Introduction

Modified purine derivatives are an important class of compounds which possess a wide spectrum of biological activities [1–6]. They are often used as antiviral, anticancer and antibacterial agents. Such intensive medicinal chemistry applications demand for constant development of novel synthetic methodologies. Frequently, the purine structure is modified in S_N_Ar reactions with N- [7–11] and S-nucleophiles [12–14] and in metal catalyzed reactions of halopurine derivatives [15–20]. Modifications of purines with O-nucleophiles are based on S_N_Ar reactions between 6-halopurine derivatives and alcohols [21–28] in the presence of a base. Alcohols are used in excess (5–40 equiv) and often play a role of both solvent and reagent. Reactions usually are performed in polar aprotic solvents such as DMF, MeCN or THF using alkoxides NaH, K_2_CO_3_ or Na_2_CO_3_ as a base, respectively.

Other methods for the introduction of alkyloxy or aryloxy substituents in the purine structure involve substitution reactions of different leaving groups such as: 1) benzotriazolyloxy group (HOBT) [8,29–32]; 2) the alkylimidazolyl group [33–34] and 3) in-situ*-*generated alkylammonia salts [35–38]. In 1995, the Robins group demonstrated S_N_Ar reactions of 6-(1,2,4-triazol-4-yl)purine with dimethylamine, sodium methoxide and sodium thiomethoxide [39]. Earlier, the use of 6-(1,2,4-triazol-1-yl)purine derivatives in S_N_Ar reactions has been reported [40]. An alternative method for the synthesis of *O*^6^-alkylpurines is Pd catalyzed C–O bond formation starting from 6-halopurines [41]. O-Alkylation of guanosine and inosine with Cu(I)-stabilized carbenes derived form α-diazocarbonyl compounds is also known [42]. Alkylation of 6-oxopurine derivatives under Mitsunobu conditions which usually proceeds with O-regioselectivity are mostly described for guanine derivatives [43–50]. In the case of C-nucleophiles there are a few precedents of transition-metal-free substitution of chloro [51–55] or 1,2,4-triazolyl [56] moieties as leaving groups at the C6 position of purine. These transformations usually require prolonged time and elevated temperatures to be completed. Among the widely studied 1,2,3-triazolyl nucleoside conjugates [57–58], the synthesis of 2-triazolylpurine derivatives containing a designed substituent at C6 has been little discussed. 6-N-Substituted purines have been the most studied [11,59–62], but 6-S- [14,63] or 6-O-analogues are less common [61].

**Scheme 1:**
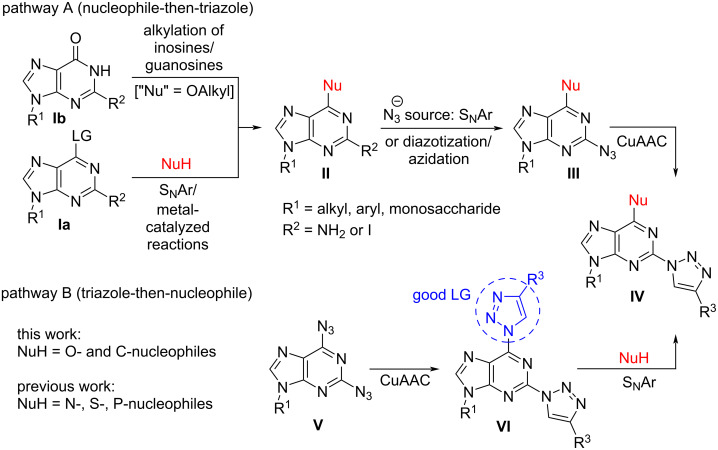
Synthetic pathways for the synthesis of 6-substituted 2-triazolylpurine derivatives **IV**.

Azolylpurine derivatives are important due to their potential as drug candidates. They can be used as agonists and antagonists of adenosine receptors [58,64–66] and against *Mycobacterium tuberculosis* [60]. They also show useful fluorescent properties [11,67–69] and can be used as metal ion sensors [70]. Therefore, it is important to develop novel methods towards this type of derivatives. To date two approaches have been used to obtain 6-substituted 2-triazolylpurine derivatives (Scheme 1). According to the pathway A, firstly a selected substituent is introduced at the C6 position of the purine ring using S_N_Ar reactions (**Ia→II**, Scheme 1). If purine contains identical leaving groups at C2 and C6 positions the reactivity order in its S_N_Ar reactions is C6 > C2 [71–72]. Also transition metal catalyzed reactions can be used for C6 functionalization of purine [73–76] or alkylation of inosine or guanosine derivatives (**Ib**→**II**, Scheme 1) [30,36]. In the next step, azide can be introduced either by a second S_N_Ar reaction on the C2-halo derivative or by diazotization/azidation at C2. Then, the Cu(I)-catalyzed azide–alkyne cycloaddition (CuAAC) reaction provides the target product **IV** (Scheme 1, pathway A) [59–61]. Pathway B is designed on the basis of our group investigations on the synthesis of 2,6-bistriazolylpurine derivatives and their application in reactions with N*-*, S*-* and P*-*nucleophiles making use of regioselective S_N_Ar reactions at C(6) (**V→VI→IV**, Scheme 1) [11,14,62–63,77–78]. The main advantage of pathway B is a straightforward access to 2,6-diazidopurines **V** and 2,6-bistriazolylpurines **VI** due to excellent nucleophilic properties of the azide ion and well-established CuAAC reaction. Pathway B also avoids performing of an S_N_Ar process on partially deactivated purines as the introduced nucleophiles are mostly seen as electron-donating substituents (e.g., R_2_N-, RS-, RO-).

Herein, we report a synthetic extension of this methodology. We have found that the pronounced leaving group character of 1,2,3-triazoles makes 2,6-bistriazolylpurines excellent substrates for S_N_Ar reactions with O- and C-nucleophiles.

## Results and Discussion

### Synthesis of 2,6-bistriazolylpurine derivatives and their reactions with O-nucleophiles

**Scheme 2:**
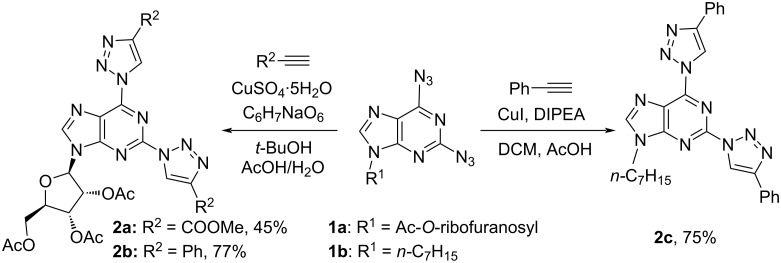
Synthesis of 2,6-bistriazolylpurine derivatives **2a–c**.

The 2,6-diazidopurine derivatives **1a** and **1b** as strategic starting materials and 2,6-bistriazolylpurine derivatives **2a–c** were obtained in the synthetic procedures developed by us before [11,14,67]. The CuAAC reaction was performed between diazide derivatives **1a** and **1b** and phenylacetylene or methyl propiolate (Scheme 2).

**Scheme 3:**
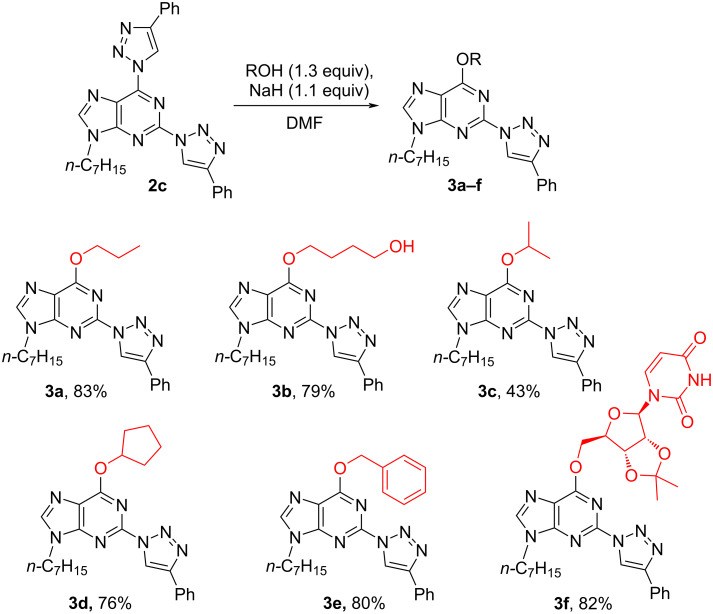
Synthesis of 6-alkyloxy-2-triazolylpurine derivatives **3a–f**.

S_N_Ar reactions between bistriazolylpurine derivatives and O-nucleophiles were first performed on N9-alkylated bistriazole **2c**. The reactions were carried out with primary and secondary alcohols in the presence of NaH in DMF. The developed transformation required only nearly equimolar loading of an alcohol and a base, and products **3a–f** were obtained in yields up to 83% (Scheme 3). In most cases the full conversion of the starting material was reached in 15–30 min at room temperature, which clearly showed the excellent leaving group ability of the triazolyl ring. These S_N_Ar reactions can also be performed in DMSO or DMF in the presence of K_2_CO_3_, but the completion of these transformations requires heating the reaction mixtures up to 60 °C for 24 h.

An S_N_Ar reaction with a non-trivial alcohol was demonstrated on the example of 2',3'-*O*-isopropylideneuridine and product **3f** was isolated after 21 h of heating at 50 °C in 82% yield. It should be noted that tertiary alcohols (e.g., *t-*BuOH) were inert in S_N_Ar reactions with 2,6-bistriazolylpurines and their attempted reactions resulted in an unidentifiable mixture of byproducts.

**Scheme 4:**
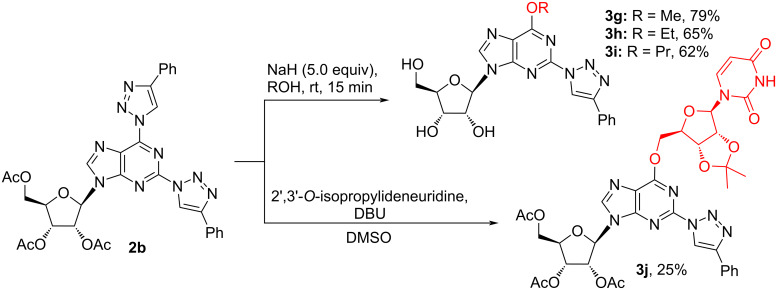
Synthesis of 6-alkyloxy-2-triazolylpurine nucleosides **3g–j**.

The following experiments were performed on 2,6-bistriazolylpurine nucleoside **2b** in MeOH, EtOH and PrOH used as solvents and nucleophiles in the presence of NaH (5.0 equiv). The excess of base and alcohol was required due to the cleavage of acetyl protecting groups. Products **3g–i** were obtained in yields of up to 79% (Scheme 4). Furthermore, purification of the products **3g–i** was complicated due to their poor solubility in organic solvents. The C6 regioselectivity of S_N_Ar reactions was proved by ^13^C NMR comparison of the products **3a–i** with similar compounds from literature [61].

Intriguingly, we were able to conserve the acetate protecting groups in product **3j**, when the S_N_Ar reaction was performed in the presence of DBU used as base. The artificial dinucleotide analogue **3j** was obtained in 25% isolated yield.

**Scheme 5:**
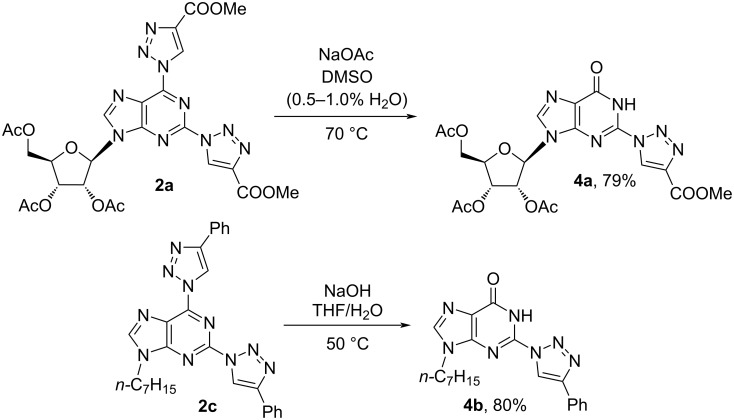
2,6-Bistriazolylpurine derivatives in S_N_Ar reactions with H_2_O/HO^−^ as nucleophiles.

We have explored also reactions of 2,6-bistriazolylpurines **2a** and **2c** with water in buffered and basic medium, respectively (Scheme 5). The buffered conditions (NaOAc/DMSO/H_2_O) were sufficiently mild to maintain the acetyl protecting groups in product **4a**. Also hydrolysis of **2c** into **4b** proceeded under mild conditions and only gentle warming to 50 °C was required.

### 2,6-Bistriazolylpurine derivatives in S_N_Ar reactions with C-nucleophiles

**Scheme 6:**
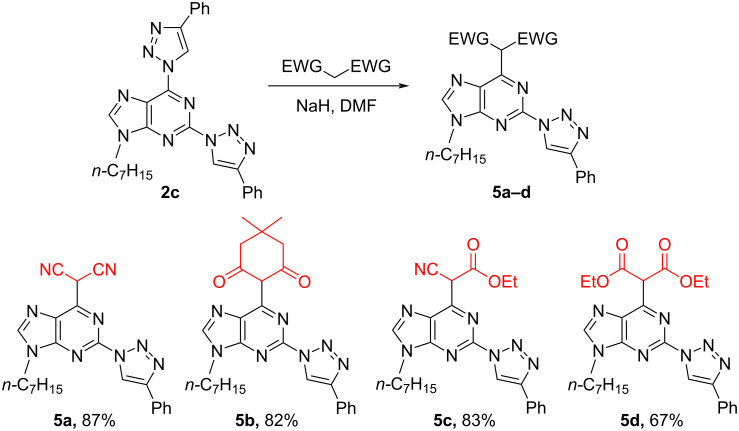
Synthesis of C6-substituted 2-triazolylpurine derivatives **5**.

Next, S_N_Ar reactions between 2,6-bistriazolylpurine **2c** and C-nucleophiles offered an easy way for the C–N bond transformation into a C–C bond. Compounds containing electron-withdrawing groups such as malonitrile, dimedone, ethyl cyanoacetate and diethyl malonate were used as C-nucleophiles. Transformations were performed in DMF in the presence of NaH and the products were obtained in high yields (Scheme 6). The lower yield of compound **5d** was obtained due to the ethyl ester hydrolysis and subsequent decarboxylation. Such side reactions were also observed for similar compounds in literature [79–80].

As a limitation of the method we have found that 2,6-bistriazolylpurine **2c** was inert to S_N_Ar reactions with deprotonated acetylacetone and diphenylmethane. Even there are reports on S_N_Ar reactions of acetylacetone with purines and pyrimidines [56,80], in our hands only polymerization of acetylacetone was observed. On the other hand, the diphenylmethane anion (p*K*_a_ 32; DMSO [81]) apparently is too basic and deprotonates purine C(8)–H, thus suspending the S_N_Ar process.

**Figure 1:**
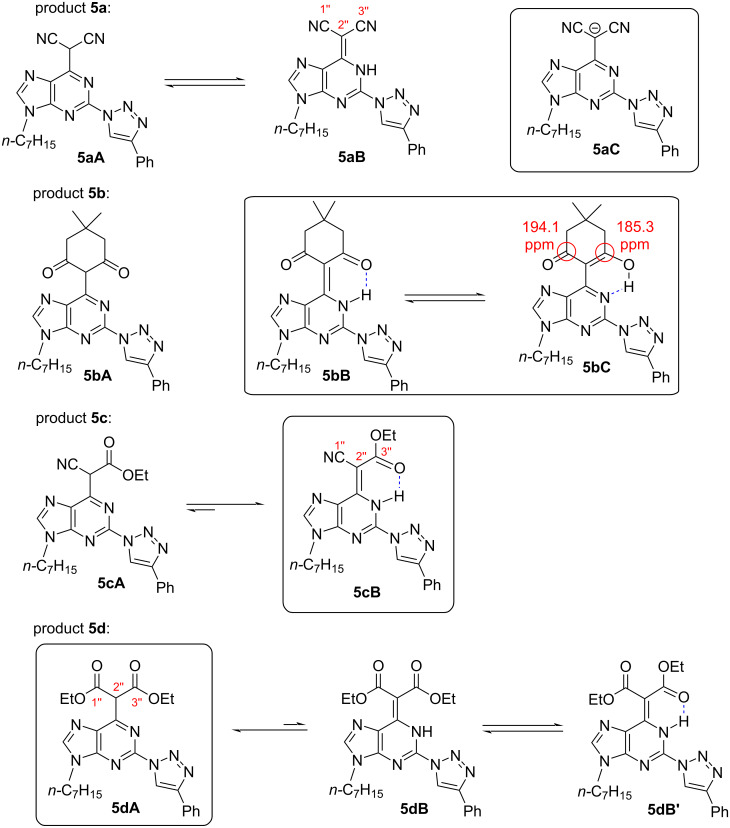
Possible tautomeric structures of compounds **5a–d**.

The structures of C6-substituted products **5a–d** were elucidated by NMR and IR analysis. These compounds can exist as either C–H acids (**A**) or N–H acids (**B**), but dimedone conjugate **5b** may possess also an enol form **C** (Figure 1).

During the structural studies of cyano group containing products **5a** and **5c** the cross signals for the C(2’’)–H system were not found using HSQC spectra, excluding the existence of C–H tautomeric forms **A**. In addition, IR analysis (KBr tablet) indicated absorption bands of cyano groups at 2205 and 2170 cm^−1^ for product **5a** and at 2205 cm^−1^ for product **5c**. These results differ from the absorption in the range of 2260–2240 cm^−1^, which would be characteristic for a cyano group attached to sp^3^-hybridized carbon [82]. On the other hand, ^13^C NMR shifts of the C(2’’) position of purine–malononitrile conjugate **5a** and ethyl cyanoacetate–purine conjugate **5c** were 40.9 and 61.7 ppm, respectively. This range does not fully correspond to the theoretical values 80–140 ppm, expected for the Csp^2^ atom of the N–H form **B**. In compound **5c** the N–H form **5cB** is possibly the major tautomer in CDCl_3_ solution as it is stabilized via an intramolecular hydrogen bond. This is supported by a smaller deviation of the C(2’’) chemical shift value (61.7 ppm) in comparison to the theoretical shifts for a Csp^2^ centre. Similar structural analogues are known in the literature [54,83–85] but their structural analysis was incomplete. As the aforementioned experiments did not determine preference for tautomer **A** or **B** of compound **5a**, it was analysed in its deprotonated form **C** (CD_3_OD/D_2_O/NaOD). Interestingly, that the ^13^C NMR spectrum of **5a** in basic medium revealed a similar chemical shift for carbon C(2’’) (40.9 ppm) as in neutral CD_3_OD.

The ^13^C NMR analysis of purine–dimedone conjugate **5b** revealed two downfield shifts of 194.1 and 185.3 ppm. It showed that the structure is not symmetrical and corresponds to either tautomer structure **B** or **C** in CDCl_3_ solution with a theoretical preference for enol form **C**. Finally, the structure of C–H tautomer **5dA** was proved by its HSQC spectrum, in which a cross peak clearly indicated the C(2’’)–H system.

## Conclusion

The S_N_Ar reactivity of 2,6-bis(1,2,3-triazol-1-yl)purine derivatives was extended with their substitution with O*-* and C-nucleophiles. The reactions proceeded under transition metal free conditions and revealed excellent C6 selectivity. The developed synthetic approach provided O-adducts with 25–83% yields and C-adducts with 67–87% yields. The methodology demonstrated the leaving group ability of the 1,2,3-triazolyl substituent at the C6 position of the purine ring.

## Experimental

### General information

^1^H and ^13^C NMR spectra were recorded with a Bruker Avance 300 or a Bruker Avance 500 spectrometer, at 300 and 75.5 MHz or 500 and 125.7 MHz, respectively. The proton signals for residual non-deuterated solvents (δ 7.26 for CDCl_3_, δ 2.50 for DMSO-*d*_6_, δ 3.31 for CD_3_OD) and the carbon signals (δ 77.1 for CDCl_3_, δ 39.5 for DMSO-*d*_6_, δ 49.0 for CD_3_OD) were used as an internal reference for ^1^H and ^13^C NMR spectra, respectively. Coupling constants are reported in Hz. Chemical shifts of signals are given in ppm and multiplicities are assigned as follows: s – singlet, d – doublet, t – triplet, m – multiplet, brs – broad singlet, tq – triplet of quartets.

Analytical thin-layer chromatography (TLC) was performed on Merck 60 Å silica gel F_254_ plates. Column chromatography was performed on Merck 40–60 µm 60 Å silica gel. Yields of products refer to chromatographically and spectroscopically homogeneous materials. The solvents used in the reactions were dried with standard drying agents and freshly distilled prior to use. Commercial reagents were used as received.

IR spectra were recorded in KBr tablets with a Perkin–Elmer Spectrum BX FTIR spectrometer (4000–450 cm^−1^). Wavelengths are given in cm^−1^.

For HPLC analysis an Agilent Technologies 1200 Series chromatograph equipped with an Agilent XDB-C18 (4.6 × 50 mm, 1.8 µm) column was used. Eluent A: 0.1% TFA solution with 5% v/v MeCN added; eluent B – MeCN. Gradient: 10–95% B 5 min, 95% B 5 min, 95–10% B 2 min. Flow: 1 mL/min. Wavelength of detection was 260 nm.

LC–MS was recorded with a Waters Acquity UPLC system equipped with Acquity UPLC BEH C18 1.7 μm, 2.1 × 50 mm; using 0.1% TFA/H_2_O and MeCN for mobile phase. HRMS analyses were performed on an Agilent 1290 Infinity series UPLC system equipped with column Extend C18 RRHD 2.1 × 50 mm, 1.8 μm connected to an Agilent 6230 TOF LC/MS mass spectrometer.

### General procedures and product characterization

Synthesis of compounds **1a,b** and **2a–c** and their characterization are described earlier [11,14,67].

### Synthesis of 6-O-substituted 2-triazolylpurines

#### General procedure A for the S_N_Ar reaction with O-nucleophiles

**9-Heptyl-2-(4-phenyl-1*H*-1,2,3-triazol-1-yl)-6-(prop-1-yl)oxy-*9H*-purine (3a):** To a suspension of 9-heptyl-2,6-bis(4-phenyl-1*H*-1,2,3-triazol-1-yl)-9*H*-purine (**2c**, 188 mg, 0.37 mmol, 1.0 equiv) in anhydrous DMF (2.5 mL) a suspension of *n*-PrOH (34 μL, 0.45 mmol, 1.2 equiv) and NaH (10 mg, 0.43 mmol, 1.2 equiv) in anhydrous DMF (0.5 mL) was added and the reaction mixture was stirred for 15 min at rt, controlled by HPLC. Then toluene or ethyl acetate (25 mL) was added to the mixture and it was extracted with 5% LiCl solution (3 × 5 mL). The organic phase was dried over anhydrous Na_2_SO_4_, filtered and evaporated. Silica gel column chromatography (DCM/MeCN 10:1) gave the product as colourless amorphous solid. Yield 115 mg, 83%. *R*_f_* =* 0.80 (DCM/MeCN 5:1); HPLC: *t*_R_ = 7.68 min, purity 98%; IR (KBr) ν (cm^−1^): 3075, 2965, 2930, 2870, 1745, 1605, 1435, 1415, 1350, 1330, 1245, 1235, 1070; ^1^H NMR (300 MHz, CDCl_3_) δ 8.70 (s, 1H, H-C(triazole)), 7.93 (s, 1H, H-C(8)), 7.91 (d, ^3^*J* = 7.6 Hz, 2H, Ar), 7.39 (t, ^3^*J* = 7.6 Hz, 2H, Ar), 7.30 (t, ^3^*J* = 7.6 Hz, 1H, Ar), 4.65 (t, ^3^*J*_1’’,2’’_ = 6.7 Hz, 2H, H_2_C(1’’)), 4.26 (t, ^3^*J*_1’,2’_ = 7.2 Hz, 2H, H_2_C(1’)), 1.96 (tq, ^3^*J*_1’’,2’’_= 6.7 Hz, ^3^*J*_2’’-3’’_= 7.4 Hz, 2H, H_2_C(2’’)), 1.93‒1.82 (m, 2H, H_2_C(2’)), 1.35‒1.26 (m, 4H, H_2_C(3’), H_2_C(4’)), 1.25‒1.17 (m, 4H, H_2_C(5’), H_2_C(6’)), 1.08 (t, ^3^*J*_2’’,3’’_= 7.4 Hz, 3H, H_3_C(3’’)), 0.81 (t, ^3^*J*_6’-7’_ = 6.9 Hz, 3H, H_3_C(7’)); ^13^C NMR (75.5 MHz, CDCl_3_) δ 161.5, 152.8, 148.3, 147.5, 143.0, 130.1, 128.7, 128.3, 125.9, 120.6, 118.6, 69.8, 44.2, 31.5, 29.9, 28.6, 26.5, 22.4, 22.1, 13.9, 10.5; HRESIMS (*m*/*z*): [M + H]^+^ calcd for C_23_H_30_N_7_O, 420.2506; found, 420.2510 (0.95 ppm).

#### General procedure B for the S_N_Ar reaction with O-nucleophiles

**9-(β-ᴅ-Ribofuranosyl)-6-methoxy-2-(4-phenyl-1*H*-1,2,3-triazol-1-yl)-9*H*-purine (3g):** To a solution of 9-(2’,3’,5’-tri-*O*-acetyl-β-ᴅ-ribofuranosyl)-2,6-bis(4-phenyl-1*H*-1,2,3-triazol-1-yl)-9*H*-purine (**2b**, 335 mg, 0.50 mmol, 1.0 equiv) in MeOH (6 mL) a suspension of NaH (60 mg, 2.52 mmol, 5.0 equiv) in MeOH (6 mL) was added and the reaction mixture was stirred for 10 min at rt, controlled by HPLC. Then AcOH (0.2 mL) was added and the mixture was partially evaporated. The suspension was centrifuged, the solids were separated and washed with MeOH (4 × 7 mL). Colourless solid. Yield 168 mg, 79%. HPLC: *t*_R_ = 4.20 min, purity 95%; IR (KBr) ν (cm^−1^): 3390, 2950, 1605, 1490, 1455, 1400, 1365, 1245, 1035, 1020; ^1^H NMR (300 MHz, DMSO-*d*_6_ + D_2_O) δ 9.38 (s, 1H, H-C(triazole)), 8.70 (s, 1H, H-C(8)), 8.02 (d, ^3^*J =* 7.6 Hz, 2H, Ar), 7.50 (t, ^3^*J =* 7.6 Hz, 2H, Ar), 7.39 (t, ^3^*J =* 7.6 Hz, 1H, Ar), 6.06 (d, ^3^*J*_1’,2’_ = 5.8 Hz, 1H, H-C(1’)), 4.65 (dd, ^3^*J*_1’,2’_ = 5.8 Hz, ^3^*J*_2’,3’_ = 4.8 Hz, 1H, H-C(2’)), 4.29 (s, 3H, (-OCH_3_)), 4.22 (dd, ^3^*J*_2’,3’_ = 4.8 Hz, ^3^*J*_3’,4’_= 3.7 Hz, 1H, H-C(3’)), 4.01 (dt, ^3^*J*_3’,4’_ = 3.7 Hz, ^3^*J*_4’,5a’_ = ^3^*J*_4’,5b’_ = 4.0 Hz, 1H, H-C(4’)), 3.71 (dd, ^3^*J*_4’,5a’_ = 4.0 Hz, ^2^*J*_5a’,5b’_ = 12.1 Hz, 1H, Ha-C(5’)), 3.60 (dd, ^3^*J*_4’,5b’_ = 4.0 Hz, ^2^*J*_5a’,5b’_ = 12.1 Hz, 1H, Hb-C(5’)); ^13^C NMR (75.5 MHz, DMSO-*d*_6_ + D_2_O) δ 161.4, 152.7, 148.0, 147.0, 143.6, 130.0, 129.2, 128.8, 125.8, 120.7, 120.5, 87.8, 86.0, 74.0, 70.4, 61.3, 55.3; HRESIMS (*m*/*z*): [M + H]^+^ calcd for C_19_H_20_N_7_O_5_, 426.1520; found, 426.1528 (1.88 ppm).

### Synthesis of C6-substituted 2-triazolylpurines

#### General procedure C for the S_N_Ar reaction with C-nucleophiles

**2-(9-Heptyl-2-(4-phenyl-1*H*-1,2,3-triazol-1-yl)-1,9-dihydro-6*H*-purin-6-ylidene)malononitrile (5a):** Under argon atmosphere to a suspension of 9-heptyl-2,6-bis(4-phenyl-1*H*-1,2,3-triazol-1-yl)-9*H*-purine (**2c**, 141 mg, 0.28 mmol, 1 equiv) in anhydrous DMF (2.5 mL) malononitrile (23 mg, 0.35 mmol, 1.3 equiv) and NaH (8 mg, 0.34 mmol, 1.2 equiv) were added and the reaction mixture was stirred for 30 min at rt, controlled by HPLC. Then ethyl acetate (25 mL) was added and the mixture was extracted with 5% LiCl solution (3 × 5 mL). The organic phase was dried over anhydrous Na_2_SO_4_, filtered and evaporated. Silica gel column chromatography (toluene/MeCN; gradient 50% → 75%) gave product **5a** as a slightly yellow amorphous solid, *R*_f_* =* 0.17 (toluene/MeCN 1:1). Yield 103 mg, 87%. HPLC: *t*_R_ = 6.33 min, purity 98%; IR (KBr) ν (cm^−1^): 3400, 2955, 2925, 2855, 2205, 2170, 1590, 1460, 1430, 1410, 1350, 1235, 1040; ^1^H NMR (300 MHz, CD_3_OD + D_2_O) δ 9.05 (s, 1H, H-C(triazole)), 8.02 (s, 1H, H-C(8)), 7.94 (d, ^3^*J* = 7.5 Hz, 2H, Ar), 7.47 (d, ^3^*J* = 7.5 Hz, 2H, Ar), 7.37 (t, ^3^*J* = 7.5 Hz, 1H, Ar), 4.28 (t, ^3^*J*_1’,2’_ = 7.2 Hz, 2H, H_2_C(1’)), 1.96‒1.83 (m, 2H, H_2_C(2’)), 1.40‒1.32 (m, 4H, H_2_C(3’), H_2_C(4’)), 1.31‒1.23 (m, 4H, H_2_C(5’), H_2_C(6’)), 0.86 (t, ^3^*J*_6’,7’_ = 6.9 Hz, 3H, H_3_C(7’)); ^13^C NMR (75.5 MHz, CD_3_OD) δ (ppm): 161.3, 150.4, 150.2, 148.7, 142.4, 131.4, 130.0, 129.5, 126.9, 125.2, 123.4, 120.7, 44.7, 40.9, 32.9, 31.2, 29.9, 27.6, 23.6, 14.3; HRESIMS (*m*/*z*)**:** [M + H]^+^ calcd for C_23_H_24_N_9_, 426.2149; found, 426.2149 (0 ppm).

## Supporting Information

File 1 Full experimental procedures and copies of ^1^H, ^13^C and ^1^H,^13^C HSQC NMR spectra.
