# Supplementary material for: 1,2,3-Triazoles as leaving groups: SNAr reactions of 2,6-bistriazolylpurines with O- and C-nucleophiles
Source: Beilstein J Org Chem. 2021 Feb 11;17:410–9. doi: 10.3762/bjoc.17.37 (PMC7884883; doi:10.3762/bjoc.17.37)
Supplement: File 1 — Full experimental procedures and copies of 1H, 13C and 1H,13C HSQC NMR spectra. [file Beilstein_J_Org_Chem-17-410-s001.pdf]

BEILSTEIN JOURNAL OF ORGANIC CHEMISTRY

Supporting Information

for

1,2,3-Triazoles as leaving groups: S_NAr reactions of 2,6-bistriazolylpurines with O- and C-nucleophiles

Dace Cīrule, Irina Novosjolova, Ērika Bizdēna and Māris Turks

Beilstein J. Org. Chem. **2021,** *17,* 410–419. doi:10.3762/bjoc.17.37

Full experimental procedures and copies of ^{1}H, ^{13}C and ^{1}H,^{13}C HSQC NMR spectra

License and Terms: This is a supporting information file under the terms of the Creative Commons Attribution License (https://creativecommons.org/licenses/by/4.0). Please note that the reuse, redistribution and reproduction in particular requires that the author(s) and source are credited and that individual graphics may be subject to special legal provisions.
The license is subject to the *Beilstein Journal of Organic Chemistry* terms and conditions: (https://www.beilstein-journals.org/bjoc/terms)

Experimental part

^{1}H and ^{13}C NMR spectra were recorded with a Bruker Avance 300 or Bruker Avance 500 spectrometer, at 300 and 75.5 MHz or 500 and 125.7 MHz, respectively. The proton signals for residual non-deuterated solvents (δ 7.26 for $CDCl_3$, δ 2.50 for DMSO-d_6, δ 3.31 for CD_3OD) and the carbon signals (δ 77.1 for $CDCl_3$, δ 39.5 for DMSO-d_6, δ 49.0 for CD_3OD) were used as an internal reference for ^{1}H and ^{13}C NMR spectra, respectively. Coupling constants are reported in Hz. Chemical shifts of signals are given in ppm and multiplicities are assigned as follows: s – singlet, d – doublet, t – triplet, m – multiplet, brs – broad singlet, tq – triplet of quartets.

Analytical thin layer chromatography (TLC) was performed on Merck 60 Å silica gel F_{254} plates. Column chromatography was performed on Merck 40–60 μm 60 Å silica gel. Yields of products refer to chromatographically and spectroscopically homogeneous materials. The solvents used in the reactions were dried with standard drying agents and freshly distilled prior to use. Commercial reagents were used as received.

IR spectra were recorded in KBr tablets with a Perkin–Elmer Spectrum BX FT-IR spectrometer (4000–450 cm^{-1}). Wavelengths are given in cm^{-1}.

For HPLC analysis an Agilent Technologies 1200 Series chromatograph equipped with an Agilent XDB-C18 (4.6 × 50 mm, 1.8 μm) column was used. Eluent A: 0.1% TFA solution with 5% v/v MeCN added; eluent B – MeCN. Gradient: 10–95% B 5 min, 95% B 5 min, 95–10% B 2 min. Flow: 1 mL/min. Wavelength of detection was 260 nm.

LC–MS was recorded with a Waters Acquity UPLC system equipped with Acquity UPLC BEH C18 1.7 μm, 2.1 × 50 mm; using 0.1% TFA/H_2O and MeCN for mobile phase. HRMS analyses were performed on an Agilent 1290 Infinity series UPLC system equipped with column Extend C18 RRHD 2.1×50 mm, 1.8 μm connected to an Agilent 6230 TOF LC/MS mass spectrometer.

GENERAL PROCEDURES AND PRODUCT CHARACTERIZATION

Synthesis of compounds **1a**,**b** and **2a–c** and their characterization are described earlier [1–3].

SYNTHESIS 6-O-SUBSTITUTED 2-TRIAZOLYLPURINE

General procedure A for S_NAr reaction with *O*-nucleophiles

9-Heptyl-2-(4-phenyl-1*H*-1,2,3-triazol-1-yl)-6-(prop-1-yl)oxy-*9H*-purine (3a)

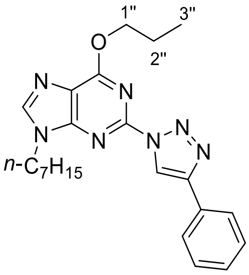

To a suspension of 9-heptyl-2,6-bis(4-phenyl-*1H*-1,2,3-triazol-1-yl)-*9H*-purine (**2c**) (188 mg, 0.37 mmol, 1 equiv) in anhydrous DMF (2.5 mL) a suspension of *n*-PrOH (34 µL, 0.45 mmol, 1.2 equiv) and NaH (10 mg, 0.43mmol, 1.2 equiv) in anhydrous DMF (0.5 mL) was added and reaction mixture was stirred for 15 min at rt, controlled by HPLC. Then toluene or ethylacetate (25 mL) was added to the mixture and it was extracted with 5% LiCl solution (3 × 5 mL). The organic phase was dried over anhydrous Na_2SO_4, filtered and evaporated. Silica gel column chromatography (DCM/MeCN = 10:1) gave product as colorless amorphous solid. Yield 115 mg, 83%. R_f = 0.80 (DCM/MeCN = 5/1). HPLC: t_R = 7.68 min, purity 98%. IR (KBr) ν (cm^{-1}): 3075, 2965, 2930, 2870, 1745, 1605, 1435, 1415, 1350, 1330, 1245, 1235, 1070. ^{1}H-NMR (300 MHz, $CDCl_3$) δ (ppm): 8.70 (s, 1H, H-C(triazole)), 7.93 (s, 1H, H-C(8)), 7.91 (d, 2H, ^3J = 7.6 Hz, Ar), 7.39 (t, 2H, ^3J = 7.6 Hz, Ar), 7.30 (t, 1H, ^3J = 7.6 Hz, Ar), 4.65 (t, 2H, $^3J_{1''-2''}$ = 6.7 Hz, H_2C(1'')), 4.26 (t, 2H, $^3J_{1'-2'}$ = 7.2 Hz, H_2C(1')), 1.96 (tq, 2H, $^3J_{1''-2''}$= 6.7 Hz, $^3J_{2''-3''}$= 7.4 Hz, H_2C(2'')), 1.93–1.82 (m, 2H, H_2C(2')), 1.35–1.26 (m, 4H, H_2C(3'), H_2C(4')), 1.25–1.17 (m, 4H, H_2C(5'), H_2C(6')), 1.08 (t, 3H, $^3J_{2''-3''}$= 7.4 Hz, H_3C(3'')), 0.81 (t, 3H, $^3J_{6'-7'}$ = 6.9 Hz, H_3C(7')). ^{13}C-NMR (75.5 MHz, $CDCl_3$) δ (ppm): 161.5, 152.8, 148.3, 147.5, 143.0, 130.1, 128.7, 128.3, 125.9, 120.6, 118.6, 69.8, 44.2, 31.5, 29.9, 28.6, 26.5, 22.4, 22.1, 13.9, 10.5. HRMS (ESI) m/z**:** $[M+H]^+$ Calcd for $C_{23}H_{30}N_7O$ 420.2506, Found 420.2510 (0.95 ppm).

9-Heptyl-2-(4-phenyl-1*H*-1,2,3-triazol-1-yl)-6-(4-hydroxybut-1-yl)oxy-*9H*-purine (3b)

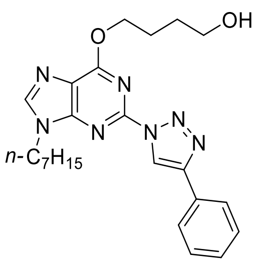

Compound **3b** was synthesized according to general procedure A: 9-heptyl-2,6-bis(4-phenyl-*1H*-1,2,3-triazol-1-yl)-*9H*-purine (**2c**) (311 mg, 0.62 mmol, 1.0 equiv), 1,4-butanediol (65 μL, ρ = 1.02. g/mL, 0.74 mmol, 1.2 equiv), NaH (17 mg, 0.70 mmol, 1.1 equiv), DMF (5 mL). Reaction conditions: 20 min, rt. Silica gel column chromatography (MeCN/toluene; gradient 20%→5%). Colorless amorphous solid, R_f = 0.38 (MeCN/toluene = 1:1). Yield 220 mg, 79%. HPLC: t_R = 8.68 min, purity 96%. IR (KBr) ν (cm^{-1}): 3375, 2925, 2855, 1605, 1460, 1440, 1410, 1345, 1245, 1230, 1015. ^{1}H-NMR (300 MHz, $CDCl_3$) δ (ppm): 8.76 (s, 1H, H-C(triazole)), 7.96 (s, 1H, H-C(8)), 7.95 (d, 2H, ^3J = 7.5 Hz, Ar), 7.45 (t, 2H, ^3J = 7.5 Hz, Ar), 7.35 (t, 1H, ^3J = 7.5 Hz, Ar), 4.79 (t, 2H, $^3J_{1''\text{-}2''}$ = 6.6 Hz, H_2C(1'')), 4.30 (t, 2H, $^3J_{1'\text{-}2'}$ = 7.2 Hz, H_2C(1')), 3.80 (t, 2H, $^3J_{3''\text{-}4''}$= 6.2 Hz, H_2C(4'')), 2.25 (brs, 1H, (-OH)), 2.09 (tt, 2H, $^3J_{1''\text{-}2''}$ = 6.6 Hz, $^3J_{2''\text{-}3''}$ = 6.4 Hz, H_2C(2'')), 2.00–1.88 (m, 2H, H_2C(2')), 1.83 (tt, 2H, $^3J_{2''\text{-}3''}$ = 6.4 Hz, $^3J_{3''\text{-}4''}$ = 6.2 Hz, H_2C(3'')), 1.41–1.30 (m, 4H, H_2C(3'), H_2C(4')), 1.30–1.20 (m, 4H, H_2C(5'), H_2C(6')), 0.85 (t, 3H, $^3J_{6'\text{-}7'}$ = 7.2 Hz, H_3C(7')). ^{13}C-NMR (75.5 MHz, $CDCl_3$) δ (ppm): 161.6, 153.0, 148.5, 147.8, 143.1, 130.2, 128.9, 128.5, 126.1, 120.7, 118.7, 68.2, 62.2, 44.4, 31.6, 30.0, 29.0, 28.7, 26.6, 25.3, 22.6, 14.1. HRMS (ESI) m/z**:** $[M+H]^+$ Calcd for $C_{24}H_{32}N_7O_2$ 450.2612, Found 450.2607 (1.11 ppm).

9-Heptyl-2-(4-phenyl-1*H*-1,2,3-triazol-1-yl)-6-(prop-2-yl)oxy-*9H*-purine (3c)

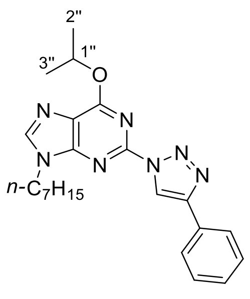

Compound **3c** was synthesized according to general procedure A: 9-heptyl-2,6-bis(4-phenyl-*1H*-1,2,3-triazol-1-yl)-*9H*-purine (**2c**) (197 mg, 0.39 mmol, 1.0 equiv), iPrOH (37 μL, ρ = 0.786 g/mL, 0.49 mmol, 1.3 equiv), NaH (11 mg, 0.47 mmol, 1.2 equiv), DMF (5 mL). Reaction conditions: 20 min, rt. C18 silica gel ($MeCN/H_2O$; gradient 33%→66%). Slightly yellow amorphous solid. R_f = 0.40 (DCM/MeCN = 10:1). Yield 70 mg, 43%. HPLC: t_R = 7.58 min, purity 95%. IR (KBr) ν (cm^{-1}): 2925, 2855, 1600, 1465, 1410, 1330, 1245, 1230, 1100, 1015. ^{1}H-NMR (300 MHz, $CDCl_3$) δ (ppm): 8.73 (s, 1H, H-C(triazole)), 7.99–7.92 (m, 3H, H-C(8), Ar), 7.45 (t, 2H, ^3J = 7.4 Hz, Ar), 7.36 (t, 1H, ^3J = 7.4 Hz, Ar), 5.81 (septet, 1H, $^3J_{1''\text{-}2''}$ = $^3J_{1''\text{-}3''}$ = 6.1 Hz, H-C(1'')), 4.31 (t, 2H, $^3J_{1'\text{-}2'}$ = 7.2 Hz, H_2C(1')), 2.01–1.86 (m, 2H, H_2C(2')), 1.55 (d, 3H, $^3J_{1''\text{-}2''}$ = 6.1 Hz, H_3C(2'')), 1.54 (d, 3H, $^3J_{1''\text{-}3''}$ = 6.1 Hz, H_3C(3''), 1.39–1.30 (m, 4H, H_2C(3'), H_2C(4')), 1.29–1.18 (m, 4H, H_2C(5'), H_2C(6')),

0.85 (t, 3H, $^3J_{6'-7'}$ = 6.7 Hz, $H_3C(7')$). ^{13}C-NMR (75.5 MHz, $CDCl_3$) δ (ppm): 161.2, 153.0, 148.6, 147.7, 142.9, 130.2, 128.9, 128.5, 126.1, 121.0, 118.7, 72.0, 44.4, 31.6, 30.0, 28.7, 26.6, 22.6, 22.0 (2C), 14.0. HRMS (ESI) m/z: $[M+H]^+$ Calcd for $C_{23}H_{30}N_7O$ 420.2506, Found 420.2526 (4.76 ppm).

6-(Cyclopentyloxy)-9-heptyl-2-(4-phenyl-1*H*-1,2,3-triazol-1-yl)-9*H*-purine (3d)

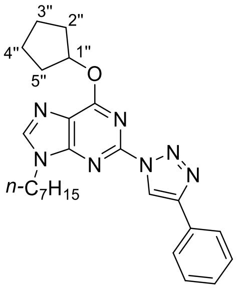

Compound **3d** was synthesized according general procedure A: 9-heptyl-2,6-bis(4-phenyl-*1H*-1,2,3-triazol-1-yl)-*9H*-purine (**2c**) (197 mg, 0.39 mmol, 1.0 equiv), cyclopentanol (45 µL, ρ = 0.949 g/mL, 0.49 mmol, 1.3 equiv), NaH (11 mg, 0.47 mmol, 1.2 equiv), DMF (5 mL). Reaction conditions: 30 min, rt. Silica gel column chromatography (toluene/MeCN = 10:1). Colorless foam. R_f = 0.35 (toluene/MeCN = 3/1). Yield 132 mg, 76%. HPLC: t_R = 8.13 min, purity 99%. IR (KBr) ν (cm^{-1}): 2955, 2930, 2855, 1600, 1465, 1440, 1410, 1345, 1230, 1015. ^{1}H-NMR (300 MHz, $CDCl_3$) δ (ppm): 8.72 (s, 1H, H-C(triazole)), 7.95–7.89 (m, 3H, H-C(8), Ar), 7.41 (t, 2H, ^3J = 7.5 Hz, Ar), 7.36– 7.27 (m, 1H, Ar), 5.90–5.81 (m, 1H, H-C(1'')), 4.28 (t, 2H, $^3J_{1''-2''}$ = 7.1 Hz, $H_2C(1')$), 2.19–1.95 (m, 4H, $H_2C(2'')$, $H_2C(5'')$, 1.95–1.79 (m, 4H, $H_2C(3'')$, $H_2C(2')$), 1.73–1.57 (m, 2H, $H_2C(4'')$), 1.37–1.27 (m, 4H, $H_2C(3')$, $H_2C(4')$), 1.26–1.16 (m, 4H, $H_2C(5')$, $H_2C(6')$), 0.81 (t, 3H, $^3J_{6'-7'}$ = 7.1 Hz, $H_3C(7')$). ^{13}C-NMR (75.5 MHz, $CDCl_3$) δ (ppm): 161.2, 152.8, 148.4, 147.6, 142.9, 130.1, 128.8, 128.4, 125.9, 120.9, 118.6, 81.2, 44.2, 32.9 (2C), 31.5, 29.9, 28.6, 26.5, 24.0 (2C), 22.5, 14.0. HRMS (ESI) m/z: $[M+H]^+$ Calcd for $C_{25}H_{32}N_7O$ 446.2663, Found 446.2676 (2.91 ppm).

6-(Benzyloxy)-9-heptyl-2-(4-phenyl-1*H*-1,2,3-triazol-1-yl)-9*H*-purine (3e)

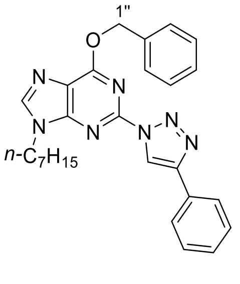

Compound **3e** was synthesized according to general procedure A: 9-heptyl-2,6-bis(4-phenyl-*1H*-1,2,3-triazol-1-yl)-*9H*-purine (**2c**) (204 mg, 0.40 mmol, 1.0 equiv), benzyl alcohol (52 µL, ρ = 1.04 g/mL, 0.51 mmol, 1.3 equiv), NaH (12 mg, 0.49 mmol, 1.2 equiv), DMF (5 mL). Reaction conditions: 20 min, rt. Silica gel column chromatography (toluene/MeCN = 12:1). Colorless amorphous solid. R_f = 0.40 (Tol/MeCN = 3/1). Yield 152 mg, 80%. HPLC: t_R = 7.86 min, purity 98%. IR (KBr) ν (cm^{-1}): 2930, 2855, 1605, 1460, 1410, 1345, 1235, 1020. ^{1}H-NMR (300 MHz, $CDCl_3$) δ (ppm): 8.72 (s, 1H, H-C(triazole)), 7.98–7.92 (m, 3H, H-C(8), Ar), 7.63 (d, 2H, ^3J = 7.3 Hz, Ar), 7.46 (t, 2H, ^3J = 7.3 Hz, Ar), 7.41–7.30 (m, 4H, Ar), 5.77 (s, 2H,

$H_2C(1'')$), 4.29 (t, 2H, $^3J_{1'-2'}$ = 7.1 Hz, $H_2C(1')$), 2.01–1.84 (m, 2H, $H_2C(2')$), 1.39–1.30 (m, 4H, $H_2C(3')$, $H_2C(4')$), 1.29–1.17 (m, 4H, $H_2C(5')$, $H_2C(6')$), 0.86 (t, 3H, $^3J_{6'-7'}$ = 7.1 Hz, $H_3C(7')$). ^{13}C-NMR (75.5 MHz, $CDCl_3$) δ (ppm): 161.1, 153.1, 148.3, 147.7, 143.3, 135.6, 130.2, 128.9, 128.8, 128.6, 128.5, 126.0 (2C), 120.8, 118.7, 69.6, 44.4, 31.6, 29.9, 28.7, 26.6, 22.5, 14.0. HRMS (ESI) m/z: $[M+H]^+$ Calcd for $C_{27}H_{30}N_7O$ 468.2506, Found 468.2476 (6.41 ppm).

9-Heptyl-2-(4-phenyl-1*H*-1,2,3-triazol-1-yl)-6-(2''',3'''-isopropylideneuridin-5'''-yl)oxy-9*H*-purine (3f)

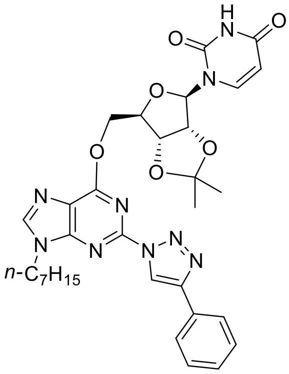

Compound **3f** was synthesized according to general procedure A: 9-heptyl-2,6-bis(4-phenyl-*1H*-1,2,3-triazol-1-yl)-*9H*-purine (**2c**) (192 mg, 0.38 mmol, 1.0 equiv), 2',3'-*O*-isopropylideneuridine (137 mg, 0.48 mmol, 1.3 equiv), NaH (11 mg, 0.46 mmol, 1.2 equiv), DMF (5 mL). Reaction conditions: 21 h, 50 °C. Silica gel column chromatography (MeCN/toluene; gradient 55%→58%). Colorless amorphous solid, R_f = 0.20 (MeCN/toluene = 2:1). Yield 200 mg, 82%. HPLC: t_R = 6.84 min, purity 97%. IR (KBr) ν (cm^{-1}): 2930, 2855, 1695, 1605, 1455, 1445, 1415, 1240, 1075, 1015. ^{1}H-NMR (300 MHz, $CDCl_3$) δ (ppm): 9.77 (s, 1H, (-NH-)), 8.77 (s, 1H, H-C(triazole)), 8.02 (s, 1H, H-C(8)), 7.93 (d, 2H, ^3J = 7.5 Hz, Ar), 7.73 (d, 1H, ^3J = 8.1 Hz, H-C(6'')), 7.42 (t, 2H, ^3J = 7.5 Hz, Ar), 7.32 (t, 1H, ^3J = 7.5 Hz, Ar), 6.01 (d, 1H, $^3J_{1'''-2'''}$= 2.2 Hz, H-C(1''')), 5.69 (d, 1H, ^3J = 8.1 Hz, H-C(5'')), 5.10 (dd, 1H, $^3J_{2'''-3'''}$= 3.6 Hz, $^3J_{3'''-4'''}$= 6.3 Hz, H-C(3''')), 5.04 (dd, 1H, $^3J_{1'''-2'''}$= 2.2 Hz, $^3J_{2'''-3'''}$= 3.6 Hz, H-C(2''')), 4.99 (dd, 1H, $^3J_{4'''-5a'''}$ = 2.6 Hz, $^2J_{5a'''-5b'''}$ = 11.9 Hz, Ha-C(5''')), 4.91 (dd, 1H, $^3J_{4'''-5b'''}$ = 3.7 Hz, $^2J_{5a'''-5b'''}$ = 11.9 Hz, Hb-C(5''')), 4.68–4.61 (m, 1H, H-C(4''')), 4.29 (t, 2H, $^3J_{1'-2'}$ = 7.1 Hz, $H_2C(1')$), 2.00–1.84 (m, 2H, $H_2C(2'')$), 1.57, 1.35 (2s, 6H, 2×($-CH_3$)),)), 1.37–1.29 (m, 4H, $H_2C(3')$, $H_2C(4')$), 1.29–1.20 (m, 4H, $H_2C(5')$, $H_2C(6')$), 0.84 (t, 3H, $^3J_{6'-7'}$ = 7.1 Hz, $H_3C(7')$). ^{13}C-NMR (75.5 MHz, $CDCl_3$) δ (ppm): 163.5, 160.5, 153.2, 150.5, 148.2, 147.7, 143.9, 141.7, 130.0, 128.7, 128.5, 126.0, 120.5, 118.7, 114.7, 103.0, 92.6, 84.7, 84.4, 80.8, 67.5, 44.5, 31.6, 29.9, 28.7, 27.2, 26.6, 25.4, 22.5, 14.0. HRMS (ESI) m/z: $[M+H]^+$ Calcd for $C_{32}H_{38}N_9O_6$ 644.2940, Found 644.2960 (3.10 ppm).

General procedure B for S_NAr reaction with *O*-nucleophiles

9-β-D-Ribofuranosyl-6-methoxy-2-(4-phenyl-1*H*-1,2,3-triazol-1-yl)-9*H*-purine (3g)

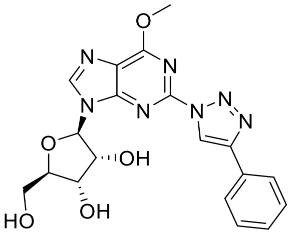

To a solution of 9-(2',3',5'-tri-*O*-acetyl-β-D-ribofuranosyl)-2,6-bis-(4-phenyl-1*H*-1,2,3-triazol-1-yl)-9*H*-purine (**2b**) (335 mg, 0.50 mmol, 1 equiv) in MeOH (6 mL) suspension of NaH (60 mg, 2.52 mmol, 5.0 equiv) in MeOH (6 mL) was added and reaction mixture was stirred for 10 min at rt, controlled by HPLC. Then AcOH (0.2 mL) was added and mixture was partially eveaporated. The suspension was centrifuged, solids were separated and washed with MeOH (4 × 7 mL). Colorless solid. Yield 168 mg, 79%. HPLC: t_R = 4.20 min, purity 95%. IR (KBr) ν (cm^{-1}): 3390, 2950, 1605, 1490, 1455, 1400, 1365, 1245, 1035, 1020. ^{1}H-NMR (300 MHz, DMSO-d_6+D_2O) δ (ppm): 9.38 (s, 1H, H-C(triazole)), 8.70 (s, 1H, H-C(8)), 8.02 (d, 2H, ^3J = 7.6 Hz, Ar), 7.50 (t, 2H, ^3J = 7.6 Hz, Ar), 7.39 (t, 1H, ^3J = 7.6 Hz, Ar), 6.06 (d, 1H, $^3J_{1'-2'}$ = 5.8 Hz, H-C(1')), 4.65 (dd, 1H, $^3J_{1'-2'}$ = 5.8 Hz, $^3J_{2'-3'}$ = 4.8 Hz, H-C(2')), 4.29 (s, 3H, (-OCH_3)), 4.22 (dd, 1H, $^3J_{2'-3'}$ = 4.8 Hz, $^3J_{3'-4'}$= 3.7 Hz, H-C(3')), 4.01 (dt, 1H, $^3J_{3'-4'}$ = 3.7 Hz, $^3J_{4'-5a'}$ = $^3J_{4'-5b'}$ = 4.0 Hz, H-C(4')), 3.71 (dd, 1H, $^3J_{4'-5a'}$ = 4.0 Hz, $^2J_{5a'-5b'}$ = 12.1 Hz, Ha-C(5')), 3.60 (dd, 1H, $^3J_{4'-5b'}$ = 4.0 Hz, $^2J_{5a'-5b'}$ = 12.1 Hz, Hb-C(5')). ^{13}C-NMR (75.5 MHz, DMSO-d_6+D_2O) δ (ppm): 161.4, 152.7, 148.0, 147.0, 143.6, 130.0, 129.2, 128.8, 125.8, 120.7, 120.5, 87.8, 86.0, 74.0, 70.4, 61.3, 55.3. HRMS (ESI) m/z**:** $[M+H]^+$ Calcd for $C_{19}H_{20}N_7O_5$ 426.1520, Found 426.1528 (1.88 ppm).

9-β-D-Ribofuranosyl-6-ethoxy-2-(4-phenyl-1*H*-1,2,3-triazol-1-yl)-9*H*-purine (3h)

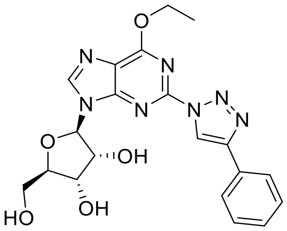

Compound **3h** was synthesized according to general procedure B: 9-(2',3',5'-tri-*O*-acetyl-β-D-ribofuranosyl)-2,6-bis-(4-phenyl-1*H*-1,2,3-triazol-1-yl)-9*H*-purine (**2b**) (357 mg, 0.54 mmol, 1.0 equiv), EtOH (12 mL), NaH (64 mg, 2.69 mmol, 5.0 equiv). Colorless solid. Yield 154 mg, 65%. HPLC: t_R = 4.54 min, purity 97%. IR (KBr) ν (cm^{-1}): 3400, 2930, 1610, 1465, 1445, 1370, 1345, 1245, 1035, 1020. ^{1}H-NMR (300 MHz, DMSO-d_6+D_2O) δ (ppm): 9.34 (s, 1H, H-C(triazole)), 8.69 (s, 1H, H-C(8)), 8.02 (d, 2H, ^3J = 7.5 Hz, Ar), 7.49 (t, 2H, ^3J = 7.5 Hz, Ar), 7.38 (t, 1H, ^3J = 7.5 Hz, Ar), 6.06 (d, 1H, $^3J_{1'-2'}$ = 5.7 Hz, H-C(1')), 4.77 (q, 2H, ^3J = 7.0 Hz, (-CH_2-)), 4.65 (dd, 1H, $^3J_{1'-2'}$ = 5.7 Hz, $^3J_{2'-3'}$ = 5.0 Hz, H-C(2')), 4.23 (dd, 1H, $^3J_{2'-3'}$ = 5.0 Hz, $^3J_{3'-4'}$= 3.4 Hz, H-C(3')), 4.01 (dt, 1H, $^3J_{3'-4'}$ = 3.4 Hz, $^3J_{4'-5a'}$ = $^3J_{4'-5b'}$ = 4.0 Hz, H-C(4')), 3.72 (dd, 1H, $^3J_{4'-5a'}$ = 4.0 Hz, $^2J_{5a'-5b'}$ = 12.0 Hz, Ha-C(5')), 3.61 (dd, 1H, $^3J_{4'-5b'}$ = 4.0 Hz,

$^{2}J_{5a'\text{-}5b'}$ = 12.0 Hz, Hb-C(5')), 1.47 (t, 3H, ^{3}J = 7.0 Hz, (-CH_3)). ^{13}C-NMR (75.5 MHz, DMSO-d_6+D_2O) δ (ppm): 161.0, 152.8, 148.0, 147.0, 143.5, 130.0, 129.2, 128.8, 125.8, 120.7, 120.4, 87.9, 86.0, 74.0, 70.4, 64.3, 61.4, 14.5. HRMS (ESI) m/z**:** $[M+H]^+$ Calcd for $C_{20}H_{22}N_7O_5$ 440.1677, Found 440.1652 (5.68 ppm).

9-β-D-Ribofuranosyl-2-(4-phenyl-1*H*-1,2,3-triazol-1-yl)-6-(prop-1-yl)oxy-9*H*-purine (3i)

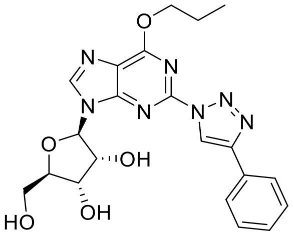

Compound **3i** was synthesized according to general procedure B: 9-(2',3',5'-tri-*O*-acetyl-β-D-ribofuranosyl)-2,6-bis-(4-phenyl-1*H*-1,2,3-triazol-1-yl)-9*H*-purine (**2b**) (386 mg, 0.58 mmol, 1.0 equiv.), *n*-PrOH (12 mL), NaH (69 mg, 2.90 mmol, 5.0 equiv.). Colorless solid. Yield 162 mg, 62%. HPLC: t_R = 4.89 min, purity 96%. IR (KBr) ν (cm^{-1}): 3410, 2935, 1610, 1485, 1465, 1365, 1245, 1035, 1020. ^{1}H-NMR (300 MHz, DMSO-d_6+D_2O) δ (ppm): 9.32 (s, 1H, H-C(triazole)), 8.68 (s, 1H, H-C(8)), 8.01 (d, 2H, ^{3}J = 7.5 Hz, Ar), 7.50 (t, 2H, ^{3}J = 7.5 Hz, Ar), 7.39 (t, 1H, ^{3}J = 7.5 Hz, Ar), 6.06 (d, 1H, $^{3}J_{1'\text{-}2'}$ = 5.7 Hz, H-C(1')), 4.68 (t, 2H, ^{3}J = 6.5 Hz, (-CH_2-)), 4.65 (dd, 1H, $^{3}J_{1'\text{-}2'}$ = 5.7 Hz, $^{3}J_{2'\text{-}3'}$ = 4.7 Hz, H-C(2')), 4.23 (dd, 1H, $^{3}J_{2'\text{-}3'}$ = 4.7 Hz, $^{3}J_{3'\text{-}4'}$= 3.7 Hz, H-C(3')), 4.01 (dt, 1H, $^{3}J_{3'\text{-}4'}$ = 3.7 Hz, $^{3}J_{4'\text{-}5a'}$ = $^{3}J_{4'\text{-}5b'}$ = 4.1 Hz, H-C(4')), 3.72 (dd, 1H, $^{3}J_{4'\text{-}5a'}$ = 4.1 Hz, $^{2}J_{5a'\text{-}5b'}$ = 12.2 Hz, Ha-C(5')), 3.61 (dd, 1H, $^{3}J_{4'\text{-}5b'}$ = 4.1 Hz, $^{2}J_{5a'\text{-}5b'}$ = 12.2 Hz, Hb-C(5')), 1.95–1.80 (m, 2H, (-CH_2-)), 1.03 (t, 3H, ^{3}J = 7.4 Hz, (-CH_3)). ^{13}C-NMR (75.5 MHz, DMSO-d_6+D_2O) δ (ppm): 161.1, 152.8, 147.9, 146.9, 143.4, 129.9, 129.1, 128.6, 125.8, 120.6, 120.3, 87.8, 86.0, 73.9, 70.4, 69.6, 61.3, 21.9, 10.4. HRMS (ESI) m/z**:** $[M+H]^+$ Calcd for $C_{21}H_{24}N_7O_5$ 454.1833, Found 454.1844 (2.42 ppm).

9-(2',3',5'-Tri-*O*-acetyl-*β*-D-ribofuranosyl)-2-(4-phenyl-1*H*-1,2,3-triazol-1-yl)-6-(2''',3'''-isopropylideneuridin-5'''-yl)oxy-9*H*-purine (3j)

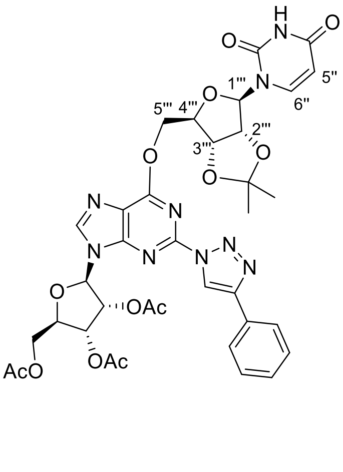

To a suspension of 9-(2',3',5'-Tri-*O*-acetyl-β-D-ribofuranosyl)-2,6-bis-(4-phenyl-1*H*-1,2,3-triazol-1-yl)-9*H*-purine (**2b**) (335 mg, 0.50 mmol, 1.0 equiv) and 2',3'-*O*-isopropylideneuridine (335 mg, 0.50 mmol, 1.0 equiv) in DMF (0.5 mL) DBU (335 mg, 0.50 mmol, 1.0 equiv) was added and reaction mixture was stirred for 2 h at rt, controlled by HPLC. Then EtOAc (25 mL) was added and mixture was extracted with 5% LiCl solution (3 × 5 mL). The

organic phase was dried over anhydrous Na_2SO_4, filtered and evaporated. Silica gel column chromatography (EtOAc/MeCN = 10:1) afforded product as colorless amorphous solid, R_f = 0.40 (EtOAc/MeCN = 10:1). Yield 169 mg, 25%. HPLC: t_R = 5.62 min, purity 96%. IR (KBr) ν (cm^{-1}): 3485, 3150, 2990, 2945, 1750, 1695, 1605, 1460, 1445, 1420, 1370, 1230, 1075, 1015. ^{1}H-NMR (300 MHz, $CDCl_3$) δ (ppm): 9.74 (brs, 1H, (-NH-)), 9.00 (s, 1H, H-C(triazole)), 8.21 (s, 1H, H-C(8)), 8.00 (d, 2H, ^3J = 7.6 Hz, Ar), 7.70 (d, 1H, ^3J = 8.2 Hz, H-C(6'')), 7.42 (t, 2H, ^3J = 7.6 Hz, Ar), 7.33 (t, 1H, ^3J = 7.6 Hz, Ar), 6.25 (d, 1H, $^3J_{1'\text{-}2'}$ = 4.1 Hz, H-C(1')), 6.05 (dd, 1H, $^3J_{1'\text{-}2'}$ = 4.1 Hz, $^3J_{2'\text{-}3'}$ = 5.5 Hz, H-C(2')), 5.99 (d, 1H, $^3J_{1'''\text{-}2'''}$= 2.1 Hz, H-C(1''')), 5.87 (t, 1H, $^3J_{2'\text{-}3'}$ = $^3J_{3'\text{-}4'}$= 5.5 Hz, H-C(3')), 5.68 (d, 1H, ^3J = 8.2 Hz, H-C(5'')), 5.12–4.92 (m, 4H, H-C(2'''), H-C(3'''), H_2C(5''')), 4.69–4.63 (m, 1H, H-C(4''')), 4.54–4.44 (m, 2H, H-C(4'), Ha-C(5')), 4.38 (dd, 1H, $^3J_{4'\text{-}5b'}$ = 5.5 Hz, $^2J_{5a'\text{-}5b'}$= 13.0 Hz, Hb-C(5')), 2.19, 2.12, 1.98 (3s, 9H, H_3CC(O)O-C(2',3',5')), 1.58, 1.36 (2s, 6H, 2×(-CH_3)). ^{13}C-NMR (75.5 MHz, $CDCl_3$) δ (ppm): 170.3, 169.7, 169.6, 163.4, 160.9, 152.4, 150.4, 148.6, 148.0, 142.8, 141.6, 129.9, 128.8, 128.5, 126.0, 121.3, 119.0, 114.6, 102.9, 92.7, 87.3, 84.7, 84.4, 80.9, 80.0, 73.3, 70.3, 67.7, 62.7, 27.2, 25.3, 20.6 (2C), 20.5. HRMS (ESI) m/z: $[M+H]^+$ Calcd for $C_{36}H_{38}N_9O_{13}$ 804.2584, Found 804.2573 (1.37 ppm).

9-(2',3',5'-Tri-*O*-acetyl-*β*-D-ribofuranosyl)-2-(4-methoxycarbonyl-1*H*-1,2,3-triazol-1-yl)- 6-oxo-1,6-dihydro-9*H*-purine (4a)

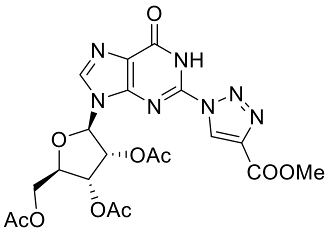

A solution of 9-(2',3',5'-tri-*O*-acetyl-*β*-D-ribofuranosyl)-2,6-bis-(4-methoxy-carbonyl-1*H*-1,2,3-triazol-1-yl)-9*H*-purine (**2a**) (150 mg, 0.24 mmol, 1.0 equiv) and sodium acetate (90 mg, 1.10 mmol, 4.6 equiv) in DMSO (5.5 mL) was stirred at 70 °C, controlled by HPLC. The reaction mixture was lyophilized and the solids were purified by silica gel column chromatography (MeOH/DCM; gradient 3% →10%, the 0.5% HCOOH was added). Colorless solid. R_f =0.63 (MeOH/DCM = 1:9 + 1% HCOOH). Yield 98 mg, 79 %. HPLC: t_R = 4.12 min, purity 99%. IR (KBr) ν (cm^{-1}): 3415, 3150, 2955, 1745, 1610,1430, 1365, 1325, 1235, 1040. ^{1}H-NMR (300 MHz, DMSO-d_6+D_2O) δ (ppm): 9.15 (s, 1H, H-C(triazole)), 8.11 (s, 1H, H-C(8)), 6.17 (d, 1H, $^3J_{1'\text{-}2'}$ = 4.9 Hz, H-C(1')), 5.99 (dd, 1H, $^3J_{1'\text{-}2'}$ = 4.9 Hz, $^3J_{2'\text{-}3'}$ = 5.9 Hz, H-C(2')), 5.73 (dd, 1H, $^3J_{2'\text{-}3'}$ = 5.9 Hz, $^3J_{3'\text{-}4'}$= 4.9 Hz, H-C(3')), 4.41 (dd, 1H, $^3J_{4'\text{-}5a'}$ = 3.6 Hz, $^2J_{5a'\text{-}5b'}$ = 11.2 Hz, Ha-C(5')), 4.38–4.32 (m, 1H, H-C(4')), 4.25 (dd, 1H, $^3J_{4'\text{-}5b'}$ = 4.9 Hz, $^2J_{5a'\text{-}5b'}$ = 11.2 Hz, Hb-C(5')), 3.87 (s, 3H, (-OCH_3)), 2.12, 2.05, 1.89 (3s, 9H, H_3CC(O)O-C(2',3',5')). ^{13}C-NMR (75.5 MHz, DMSO-d_6+D_2O) δ (ppm): 170.1, 169.7,

169.5, 166.6, 160.8, 149.7, 149.6, 138.5, 138.1, 127.1, 124.2, 85.8, 79.3, 72.3, 70.2, 62.8, 52.0, 20.44, 20.37, 20.3. HRMS (ESI) m/z: $[M+H]^+$ Calcd for $C_{20}H_{22}N_7O_{10}$ 520.1423, Found 520.1430 (1.35 ppm).

9-Heptyl-2-(4-phenyl-1*H*-1,2,3-triazol-1-yl)-1,9-dihydro-6*H*-purin-6-one (4b)

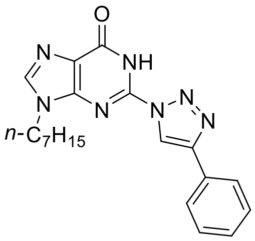

To a solution of 9-heptyl-2,6-bis(4-phenyl-*1H*-1,2,3-triazol-1-il)-*9H*-purine (**2c**) (184 mg, 0.37 mmol, 1.0 equiv.) in THF (2.5 mL) 1M NaOH aqueous solution (0.5 mL, 0.50 mmol, 1.4 equiv) was added and stirred for 8 h at 50 ^oC, controlled by HPLC. Additionally, 1 M NaOH aqueous solution (0.5 mL, 0.50 mmol, 1.4 equiv) was added and reaction mixture was stirred for 4 h at 70 ^oC, controlled by HPLC. The reaction was neutralized with 10% AcOH solution and evaporated under pressure and purified by silica gel column chromatography (DCM/MeOH = 20:1 + 0.5% HCOOH). Colorless amorphous slid, R_f = 0.40 (DCM/MeOH = 20:1). Yield 110 mg, 80%. HPLC: t_R = 6.39 min, purity 96%. IR (KBr) ν (cm^{-1}): 3435, 3100, 2930, 2855, 1715, 1625, 1595, 1455, 1440, 1410, 1360, 1235,1015. ^{1}H-NMR (300 MHz, DMSO-d_6+D_2O) δ (ppm): 9.29 (s, 1H, H-C(triazole)), 8.28 (s, 1H, H-C(8)), 8.01 (d, 2H, ^3J = 7.4 Hz, Ar), 7.50 (t, 2H, ^3J = 7.4 Hz, Ar), 7.41 (t, 1H, ^3J = 7.4 Hz, Ar), 4.21 (t, 2H, $^3J_{1'-2'}$ = 6.9 Hz, $H_2C(1')$), 1.85 (quintet, 2H, $^3J_{1'-2'}$ = $^3J_{2'-3'}$ = 6.9 Hz, $H_2C(2')$), 1.34–1.12 (m, 8H, $H_2C(3')$, $H_2C(4')$, $H_2C(5')$, $H_2C(6')$), 0.79 (t, 3H, $^3J_{6'-7'}$ = 6.9 Hz, $H_3C(7')$). ^{13}C-NMR (75.5 MHz, DMSO-d_6) δ (ppm): 158.4, 149.1, 146.9, 144.5, 142.2, 129.5, 128.9, 128.5, 125.6, 121.7, 119.9, 43.3, 31.1, 29.2, 28.0, 25.8, 21.9, 13.8. HRMS (ESI) m/z: $[M+H]^+$ Calcd for $C_{20}H_{24}N_7O$ 378.2037, Found 378.2053 (4.23 ppm).

SYNTHESIS OF 6-*C*-SUBSTITUTED 2-TRIAZOLYLPURINES

General procedure C for S_NAr reaction with *C*-nucleophiles

2-(9-Heptyl-2-(4-phenyl-1*H*-1,2,3-triazol-1-yl)-1,9-dihydro-6*H*-purin-6-ylidene)malononitrile (5a)

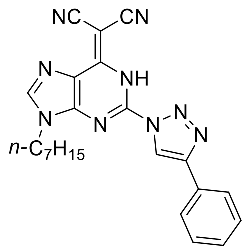

Under argon atmosphere to a suspension of 9-heptyl-2,6-bis(4-phenyl-*1H*-1,2,3-triazol-1-yl)-*9H*-purine (**2c**) (141 mg, 0.28 mmol, 1 equiv.) in anhydrous DMF (2.5 mL) malononitrile (23 mg, 0.35 mmol, 1.3 equiv) and NaH (8 mg, 0.34 mmol, 1.2 equiv) were added and reaction mixture was stirred for 30 min at rt, controlled by HPLC. Then ethylacetate (25 mL) was added and mixture was extracted with 5%

LiCl solution (3 × 5 mL). The organic phase was dried over anhydrous Na_2SO_4, filtered and evaporated. Silica gel column chromatography (toluene/MeCN; gradient 50% →75%) gave product **5a** as a slightly yellow amorphous solid, R_f = 0.17 (toluene/MeCN = 1:1). Yield 103 mg, 87%. HPLC: t_R = 6.33 min, purity 98%. IR (KBr) ν (cm^{-1}): 3400, 2955, 2925, 2855, 2205, 2170, 1590, 1460, 1430, 1410, 1350, 1235, 1040. ^{1}H-NMR (300 MHz, CD_3OD+D_2O) δ (ppm): 9.05 (s, 1H, H-C(triazole)), 8.02 (s, 1H, H-C(8)), 7.94 (d, 2H, ^3J = 7.5 Hz , Ar), 7.47 (d, 2H, ^3J = 7.5 Hz, Ar), 7.37 (t, 1H, ^3J = 7.5 Hz, Ar), 4.28 (t, 2H, $^3J_{1'-2'}$ = 7.2 Hz, $H_2C(1')$), 1.96–1.83 (m, 2H, $H_2C(2')$), 1.40–1.32 (m, 4H, $H_2C(3')$, $H_2C(4')$), 1.31–1.23 (m, 4H, $H_2C(5')$, $H_2C(6')$), 0.86 (t, 3H, $^3J_{6'-7'}$ = 6.9 Hz, $H_3C(7')$). ^{13}C-NMR (75.5 MHz, CD_3OD) δ (ppm): 161.3, 150.4, 150.2, 148.7, 142.4, 131.4, 130.0, 129.5, 126.9, 125.2, 123.4 (2C)[1], 120.7, 44.7, 40.9, 32.9, 31.2, 29.9, 27.6, 23.6, 14.3. HRMS (ESI) m/z: $[M+H]^+$ Calcd for $C_{23}H_{24}N_9$ 426.2149, Found 426.2149 (0 ppm).

2-(9-Heptyl-2-(4-phenyl-1*H*-1,2,3-triazol-1-yl)-9*H*-purin-6-yl)-3-hydroxy-5,5-dimethylcyclohex-2-en-1-one (5b)

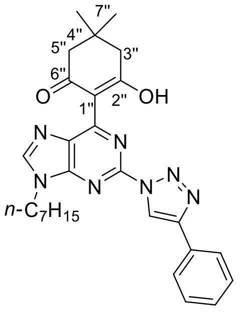

Compound **5b** was synthesized according to general procedure C: 9-heptyl-2,6-bis(4-phenyl-*1H*-1,2,3-triazol-1-yl)-*9H*-purine (**2c**) (213 mg, 0.42 mmol, 1.0 equiv), dimedone (71 mg, 0.51 mmol, 1.2 equiv), NaH (12 mg, 0.51 mmol, 1.2 equiv), DMF (2 mL). Reaction conditions: 16 h 60 °C. Silica gel column chromatography (DCM/MeOH = 20:1). Colorless solid, R_f = 0.22 (DCM/MeOH = 20/1). Yield 173 mg, 82%. HPLC: t_R = 6.79 min, purity 96%. IR (KBr) ν (cm^{-1}): 3410, 3080, 2955, 2930, 2860, 1670, 1585, 1445, 1410, 1320, 1235, 1010. ^{1}H-NMR (300 MHz, $CDCl_3$) δ (ppm): 16.17 (brs, 1H, (-OH)), 8.76 (s, 1H, H-C(triazole)), 8.14 (s, 1H, H-C(8)), 7.96 (d, 2H, ^3J = 7.5 Hz, Ar), 7.47 (d, 2H, ^3J = 7.5 Hz, Ar), 7.38 (t, 1H, ^3J = 7.5 Hz, Ar), 4.29 (t, 2H, $^3J_{1'-2'}$ = 7.1 Hz, $H_2C(1')$), 2.75–2.50 (m, 4H, $H_2C(3'')$, $H_2C(5'')$), 2.01–1.87 (m, 2H, $H_2C(2')$), 1.44–1.34 (m, 4H, $H_2C(3')$, $H_2C(4')$), 1.33–1.22 (m, 4H, $H_2C(5')$, $H_2C(6')$), 1.19 (s, 6H, 2 × $H_3C(7'')$), 0.87 (t, 3H, $^3J_{6'-7'}$ = 7.1 Hz, $H_3C(7')$). ^{13}C-NMR (75.5 MHz, $CDCl_3$) δ (ppm): 194.1, 185.3, 154.6, 152.8, 148.3, 146.0, 144.1, 129.8, 129.0, 128.8, 128.2, 126.1, 117.6, 108.8, 52.0, 45.5, 44.2, 31.64, 31.56, 30.0, 28.7, 28.5, 26.7, 22.6, 14.1. HRMS (ESI) m/z: $[M+H]^+$ Calcd for $C_{28}H_{34}N_7O_2$ 500.2768, Found 500.2790 (4.40 ppm).

[1] Determined, using HMBC spectra.

Ethyl 2-cyano-2-(9-heptyl-2-(4-phenyl-1H-1,2,3-triazol-1-yl)-1,9-dihydro-6*H*-purin-6-ylidene)acetate (5c)

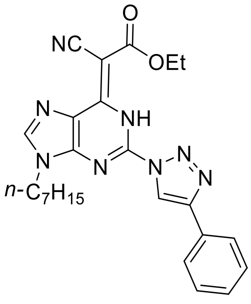

Compound **5c** was synthesized according general procedure C: 9-heptyl-2,6-bis(4-phenyl-*1H*-1,2,3-triazol-1-yl)-*9H*-purine (**2c**) (201 mg, 0.40 mmol, 1.0 equiv), ethyl cyanoacetate (51 µL, ρ = 1.063 g/mL, 0.48 mmol, 1.2 equiv), NaH (19 mg, 0.48 mmol, 1.2 equiv), DMF (2 mL). Reaction conditions: 1 h, rt. Silica gel column chromatography (DCM/MeOH = 100:1). Colorless amorphous solid. R_f = 0.40 (DCM/MeOH = 20/1). Yield 156 mg, 83%. HPLC: t_R = 7.49 min, purity 96%. IR (KBr) ν (cm^{-1}): 3420, 3090, 2925, 2855, 2205, 1640, 1565, 1475, 1300, 1230, 1000. ^{1}H-NMR (300 MHz, 50 °C, $CDCl_3$) δ (ppm): 15.34 (brs, 1H, (-NH)), 8.67 (s, 1H, H-C(triazole)), 7.97 (s, 1H, H-C(8)), 7.96 (d, 2H, ^3J = 7.6 Hz, Ar), 7.54–7.39 (m, 3H, Ar), 4.41 (q, 2H, ^3J = 7.1 Hz, (-CH_2-)), 4.25 (t, 2H, $^3J_{1'-2'}$ = 7.1 Hz, H_2C(1')), 1.95 (quintet, 2H, $^3J_{1'-2'}$ = $^3J_{2'-3'}$ = 7.1 Hz, H_2C(2')), 1.47–1.25 (m, 11H, H_2C(3'), H_2C(4'), H_2C(5'), H_2C(6'), (-CH_3)), 0.91 (t, 3H, $^3J_{6'-7'}$ = 7.1 Hz, H_3C(7')). ^{13}C-NMR (75.5 MHz, 50 °C, $CDCl_3$) δ (ppm): 169.8, 150.0, 149.6, 145.5, 141.8, 140.8, 129.5, 129.25, 129.17, 126.4, 122.6, 117.1, 116.8, 61.73, 61.69, 44.5, 31.7, 30.3, 28.8, 26.7, 22.7, 14.5, 14.1. HRMS (ESI) m/z**:** $[M+H]^+$ Calcd for $C_{25}H_{29}N_8O_2$ 473.2408, Found 473.2427 (4.01 ppm).

Diethyl 2-(9-heptyl-2-(4-phenyl-1*H*-1,2,3-triazol-1-yl)-9*H*-purin-6-yl)malonate (5d)

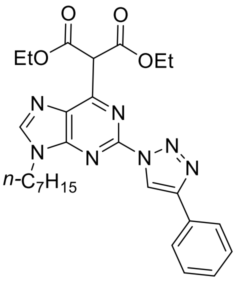

Compound **5d** was synthesized according to general procedure C: 9-heptyl-2,6-bis(4-phenyl-*1H*-1,2,3-triazol-1-yl)-*9H*-purine (**2c**) (244 mg, 0.48 mmol, 1.0 equiv), diethyl malonate (87 µL, ρ = 1.05 g/mL, 0.58 mmol, 1.2 equiv), NaH (23 mg, 0.58 mmol, 1.2 equiv), DMF (3 mL). Reaction conditions: 1 h, rt. Silica gel column chromatography (toluene/MeCN; gradient 5% →7%). Colorless amorphous solid, R_f = 0.56 (toluene/MeCN = 3:1). Yield 168 mg, 67%. HPLC: t_R = 7.43 min, purity 98%. IR (KBr) ν (cm^{-1}): 2925, 2860, 1750, 1735, 1605, 1470, 1315, 1235, 1015. ^{1}H-NMR (500 MHz, $CDCl_3$) δ (ppm): 8.81 (s, 1H, H-C(triazole)), 8.15 (s, 1H, H-C(8)), 7.96 (d, 2H, ^3J = 7.6 Hz, Ar), 7.47 (d, 2H, ^3J = 7.6 Hz, Ar), 7.38 (t, 1H, ^3J = 7.6 Hz, Ar), 5.53 (s, 1H, H-C(2'')), 4.38 (t, 2H, ^3J = 7.3 Hz, H_2C(1')), 4.34 (t, 4H, ^3J = 7.2 Hz, 2 × (-CH_2-)), 1.99 (quintet, 2H, ^3J = 7.3 Hz, H_2C(2')), 1.42–1.35 (m, 4H, 2

× (-CH_2-)), 1.31 (t, 6H, ^3J= 7.2 Hz, 2 × (-CH_3)), 1.30–1.24 (m, 4H, 2 × (-CH_2-)), 0.87 (t, 3H, $^3J_{6'-7'}$ = 6.7 Hz, H_3C(7')). ^{13}C-NMR (125.7 MHz, $CDCl_3$) δ (ppm): 166.0, 154.0, 153.2, 149.0, 148.0, 146.4, 132.6, 130.2, 129.0, 128.7, 126.2, 119.1, 62.6, 56.4, 44.6, 31.7, 30.0, 28.8, 26.8, 22.7, 14.2, 14.1. HRMS (ESI) m/z**:** $[M+H]^+$ Calcd for $C_{27}H_{34}N_7O_4$ 520.2667, Found 520.2688 (4.04 ppm).

Copies of ^{1}H, ^{13}C and ^{1}H-^{13}C HSQC NMR spectra

9-Heptyl-2-(4-phenyl-1*H*-1,2,3-triazol-1-yl)-6-(prop-1-yl)oxy-*9H*-purine (3a)

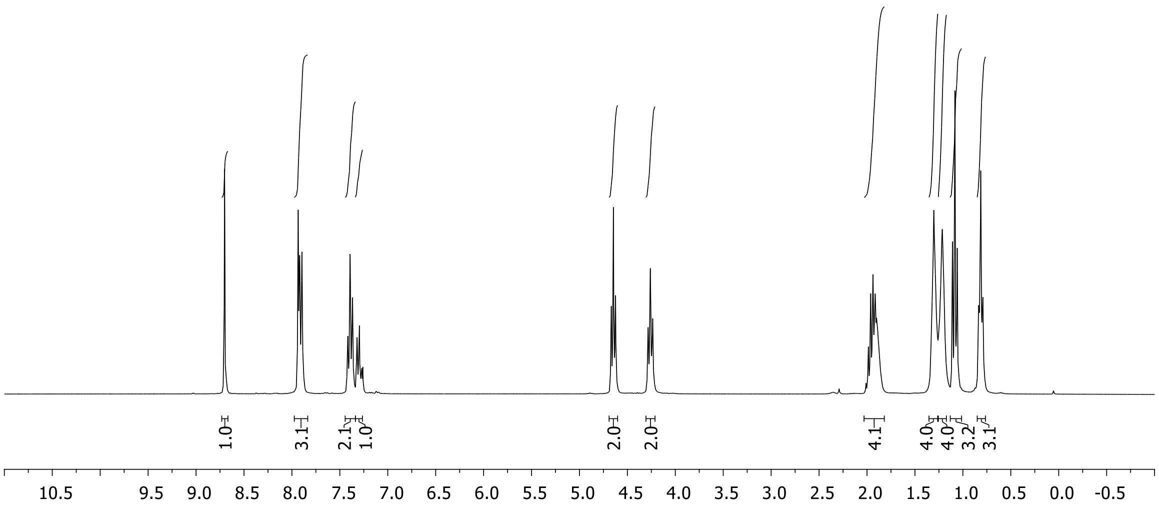

Figure S1: ^{1}H-NMR (300 MHz, $CDCl_3$) spectrum.

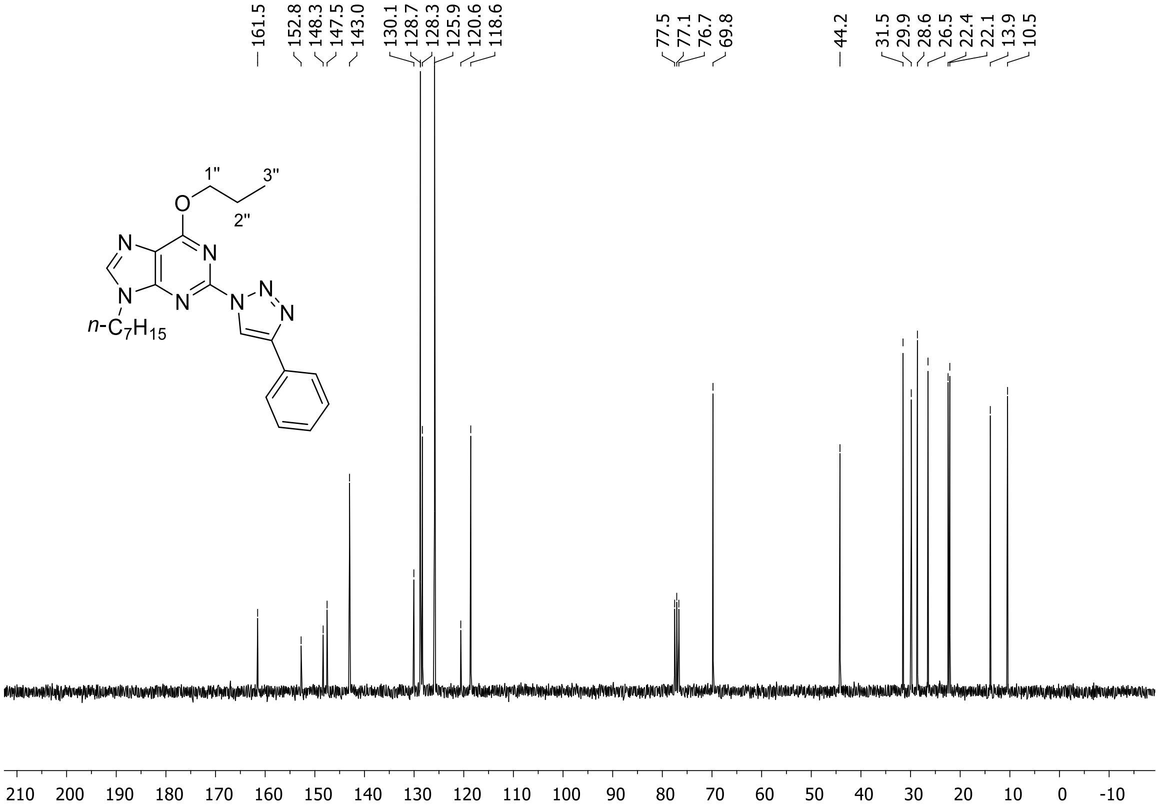

Figure S2: ^{13}C-NMR (75.5 MHz, $CDCl_3$) spectrum.

9-Heptyl-2-(4-phenyl-1*H*-1,2,3-triazol-1-yl)-6-(4-hydroxybut-1-yl)oxy-*9H*-purine (3b)

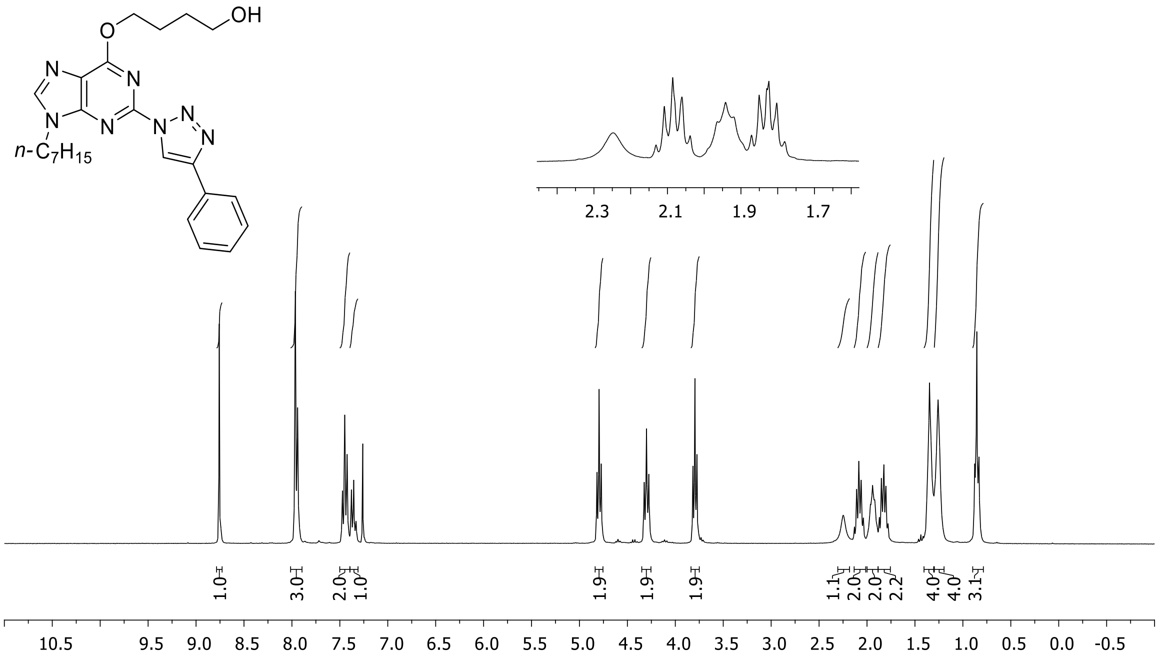

Figure S3: ^{1}H-NMR (300 MHz, $CDCl_3$) spectrum.

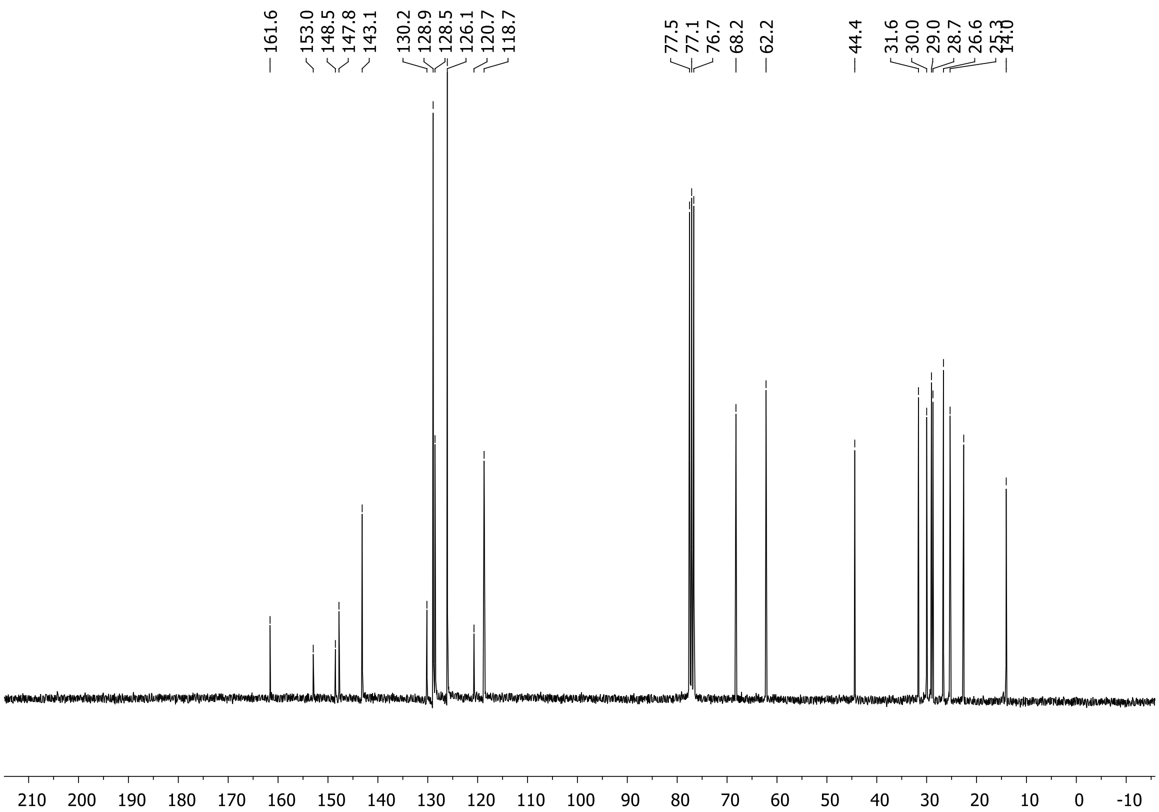

Figure S4: ^{13}C-NMR (75.5 MHz, $CDCl_3$) spectrum.

9-Heptyl-2-(4-phenyl-1*H*-1,2,3-triazol-1-yl)-6-(prop-2-yl)oxy-*9H*-purine (3c)

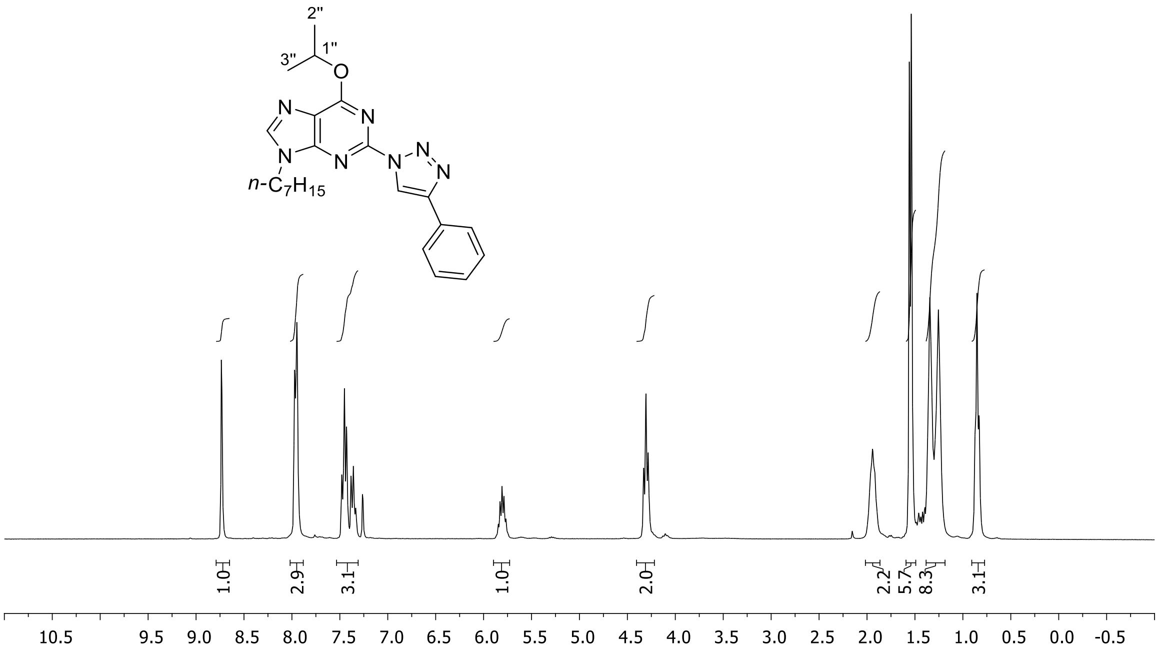

Figure S5: ^{1}H-NMR (300 MHz, $CDCl_3$) spectrum.

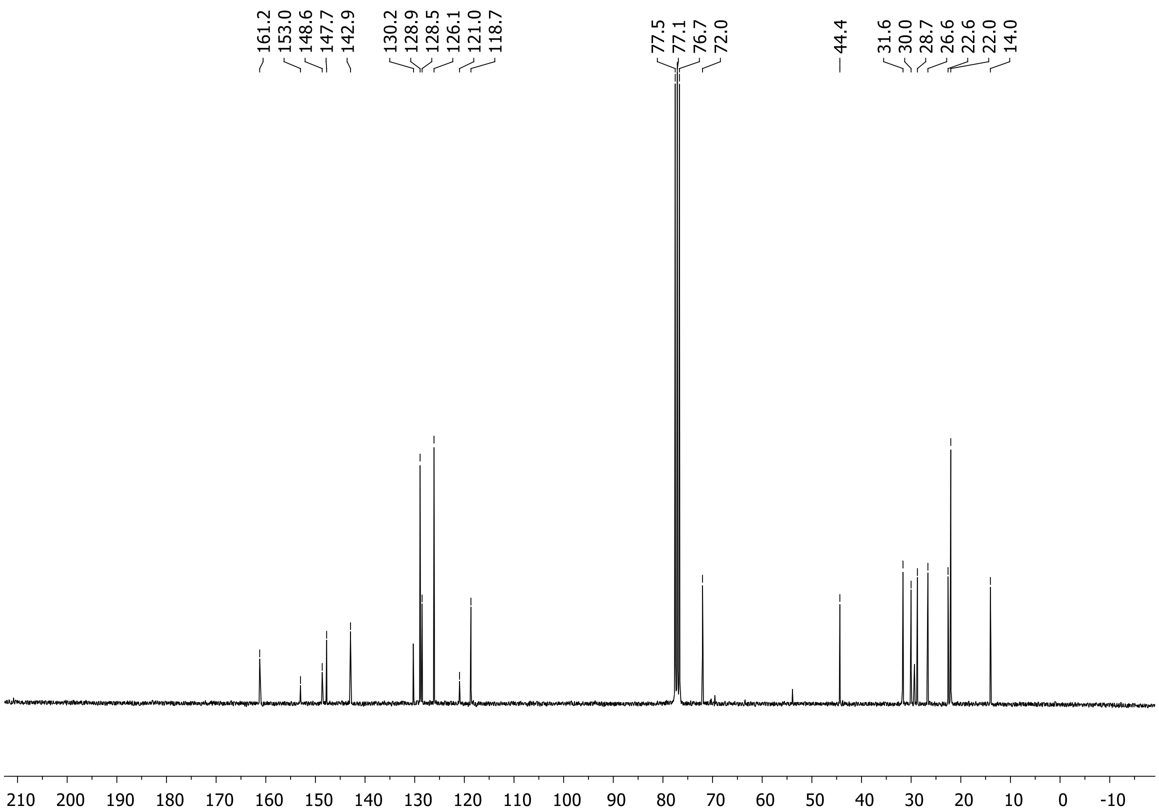

Figure S6: ^{13}C-NMR (75.5 MHz, $CDCl_3$) spectrum.

6-(Cyclopentyloxy)-9-heptyl-2-(4-phenyl-1*H*-1,2,3-triazol-1-yl)-9*H*-purine (3d)

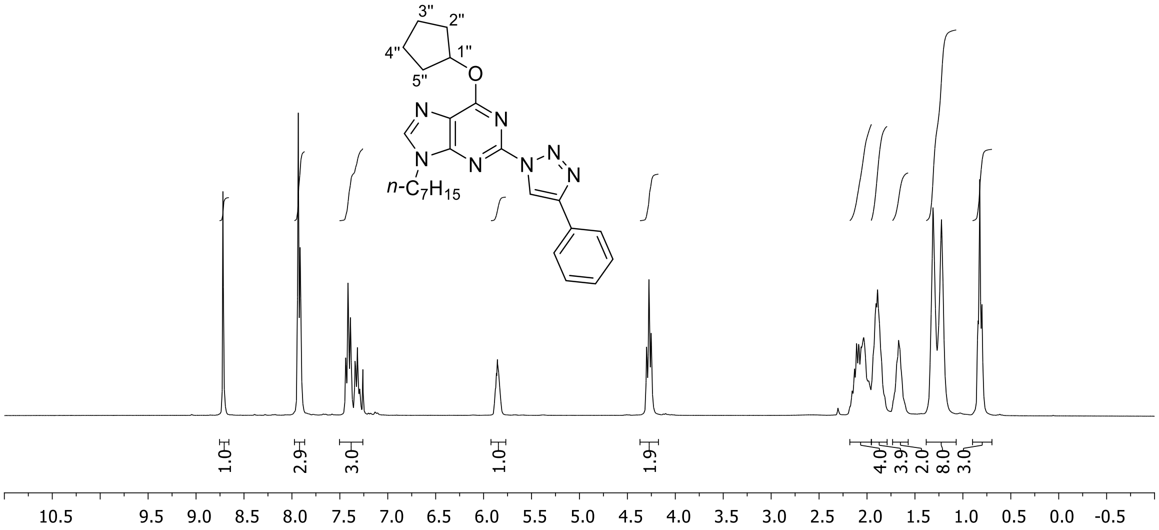

Figure S7: ^{1}H-NMR (300 MHz, $CDCl_3$) spectrum.

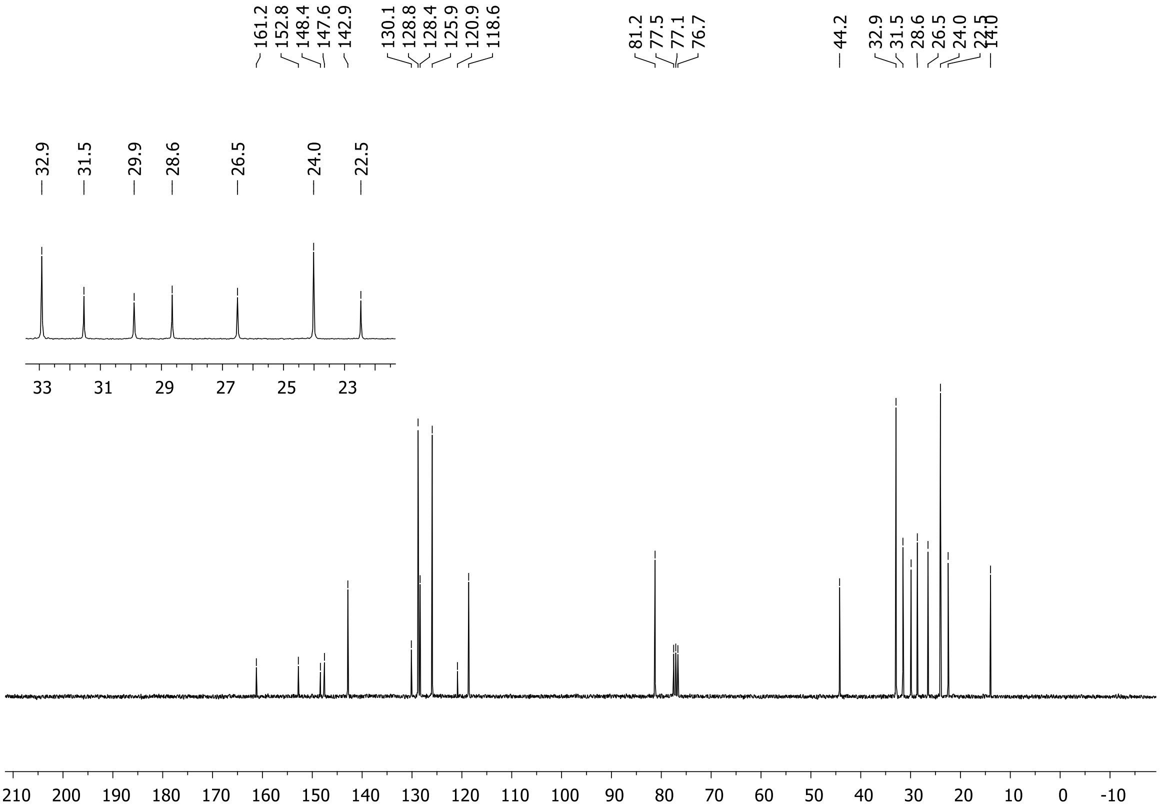

Figure S8: ^{13}C-NMR (75.5 MHz, $CDCl_3$) spectrum.

6-(Benzyloxy)-9-heptyl-2-(4-phenyl-1*H*-1,2,3-triazol-1-yl)-9*H*-purine (3e)

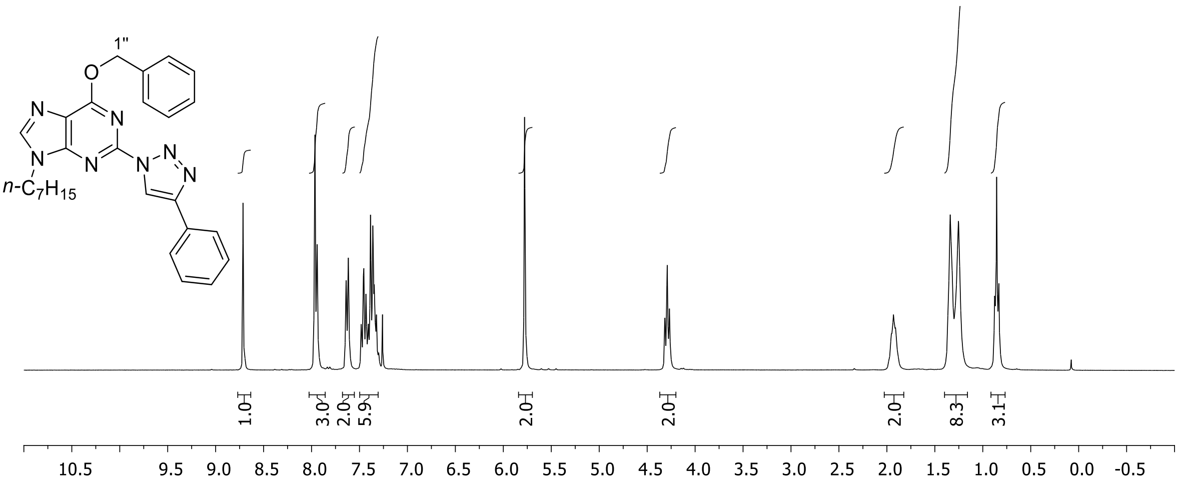

Figure S9: ^{1}H-NMR (300 MHz, $CDCl_3$) spectrum.

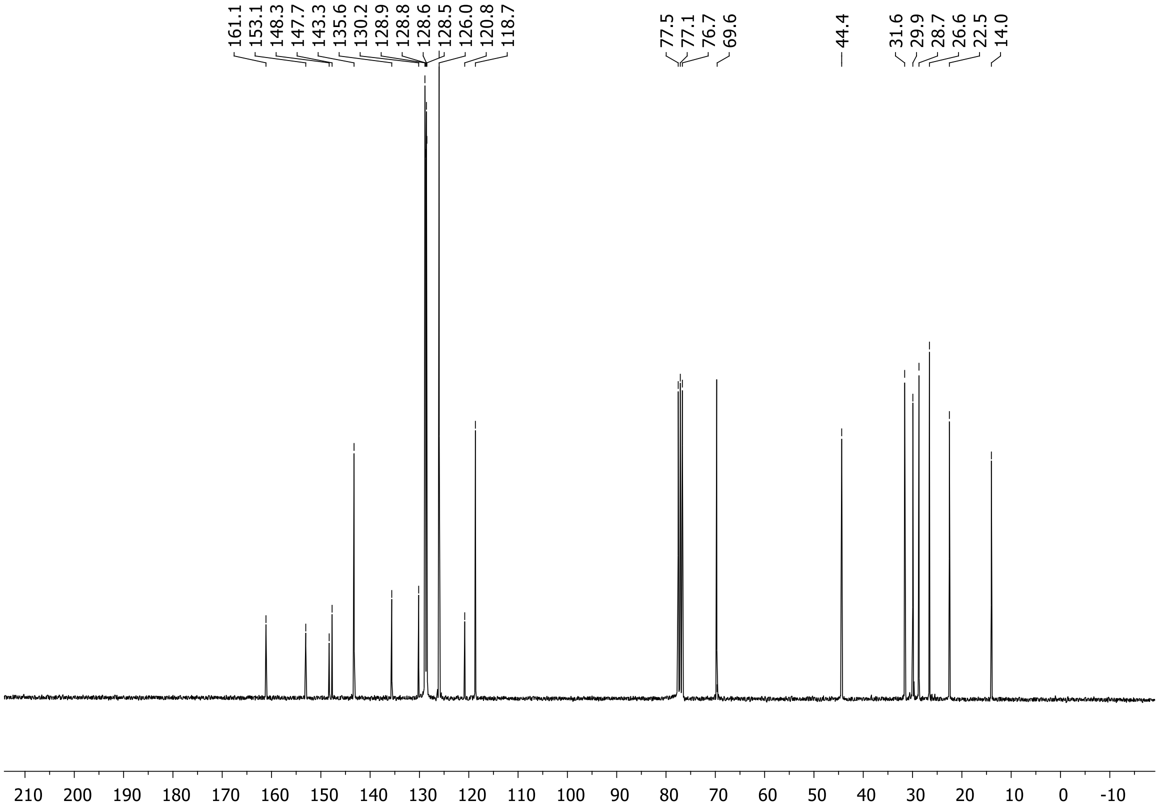

Figure S10: ^{13}C-NMR (75.5 MHz, $CDCl_3$) spectrum.

9-Heptyl-2-(4-phenyl-1*H*-1,2,3-triazol-1-yl)-6-(2''',3'''-isopropylideneuridin-5'''-yl)oxy-9*H*-purine (3f)

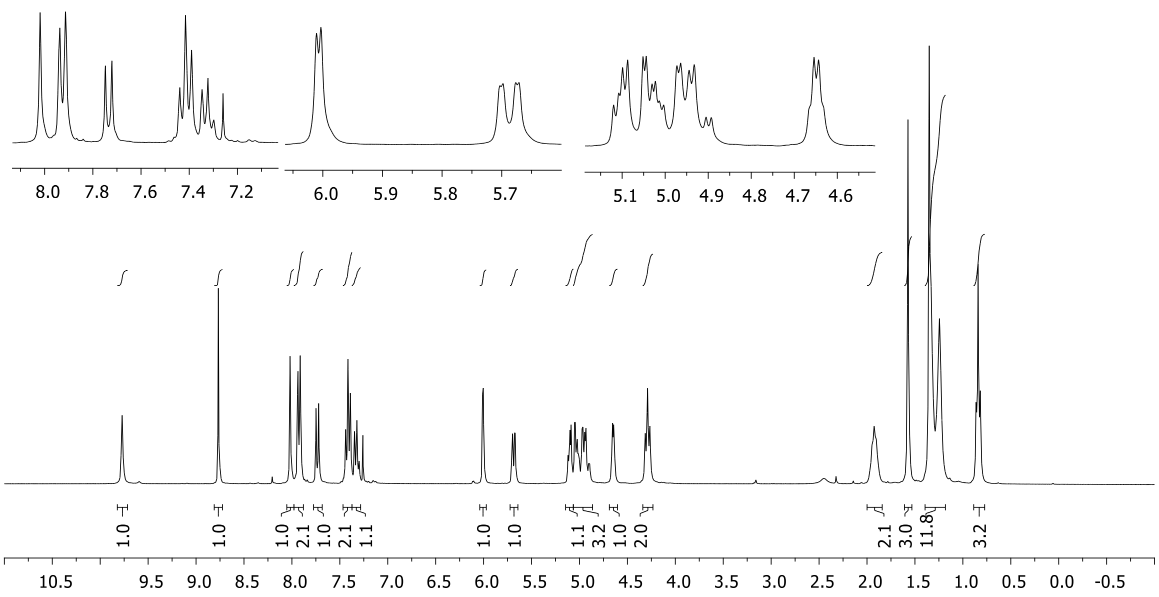

Figure S11: ^{1}H-NMR (300 MHz, $CDCl_3$) spectrum.

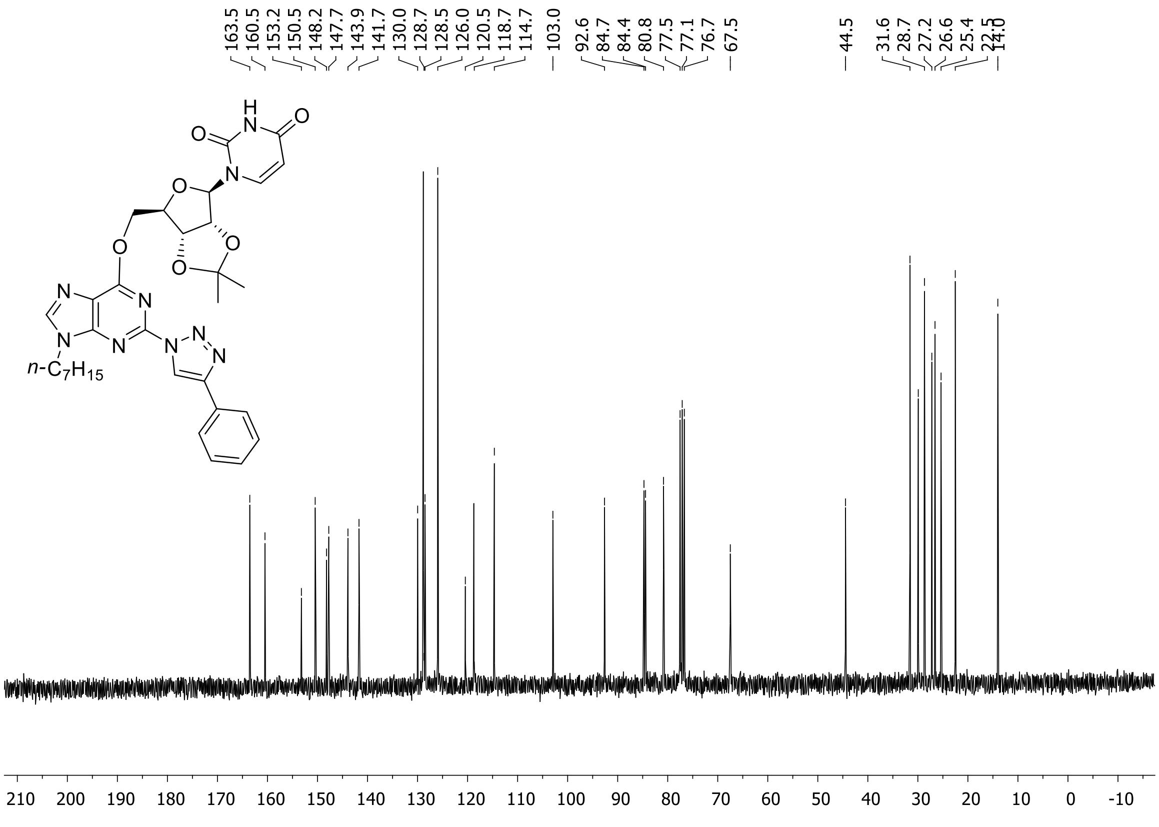

Figure S12: ^{13}C-NMR (75.5 MHz, $CDCl_3$) spectrum.

9-β-D-Ribofuranosyl-6-methoxy-2-(4-phenyl-1*H*-1,2,3-triazol-1-yl)-9*H*-purine (3g)

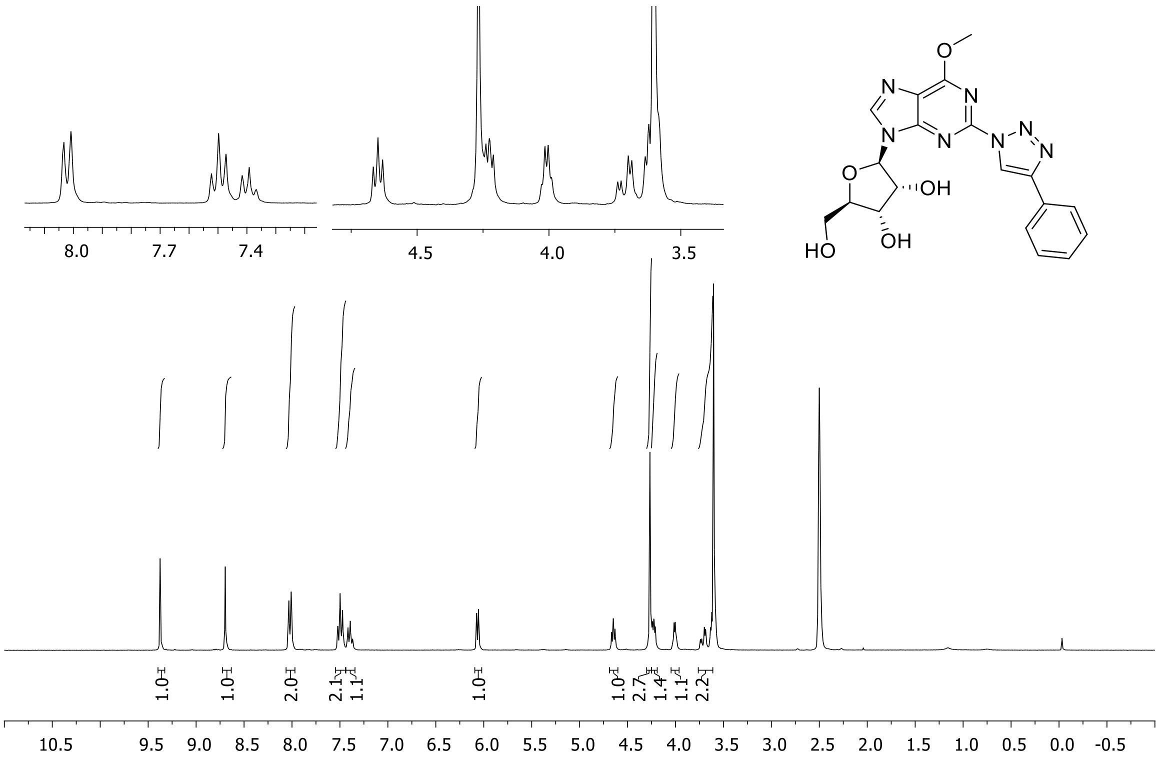

Figure S13: ^{1}H-NMR (300 MHz, DMSO-d_6+D_2O) spectrum.

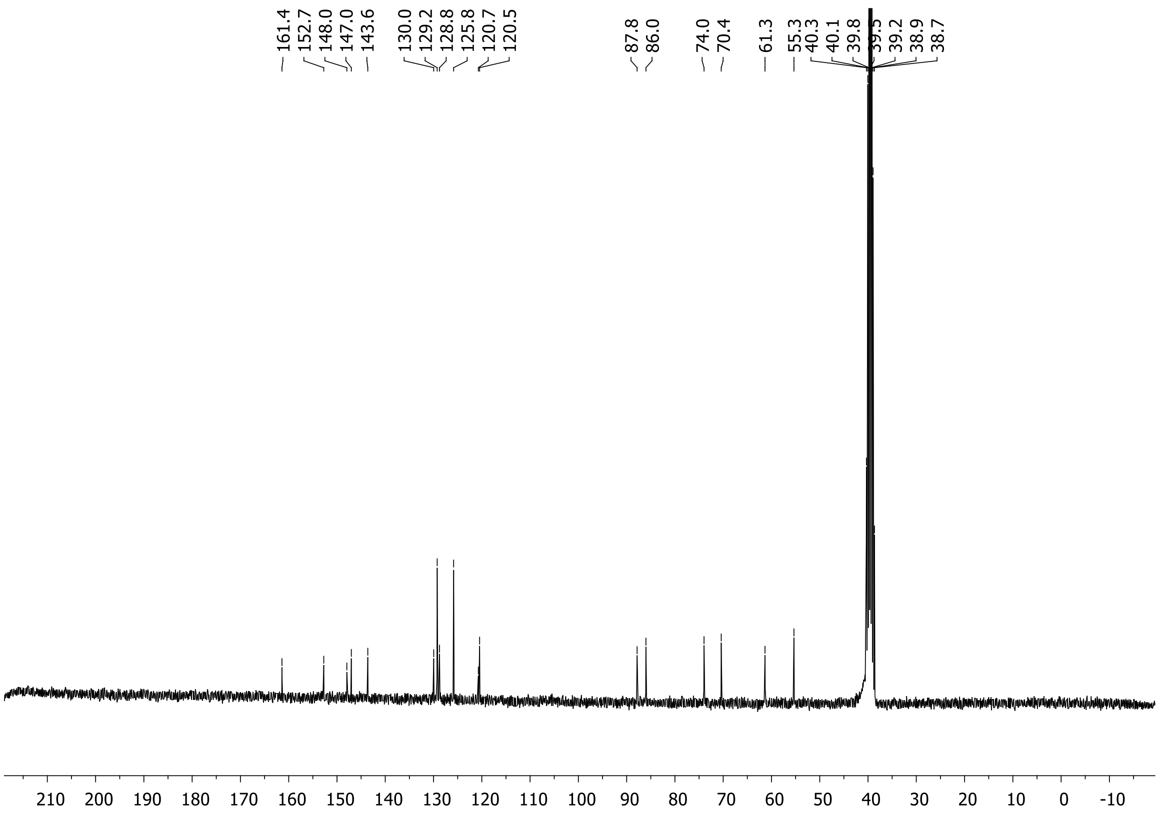

Figure S14: ^{13}C-NMR (75.5 MHz, DMSO-d_6+D_2O) spectrum.

9-β-D-Ribofuranosyl-6-ethoxy-2-(4-phenyl-1*H*-1,2,3-triazol-1-yl)-9*H*-purine (3h)

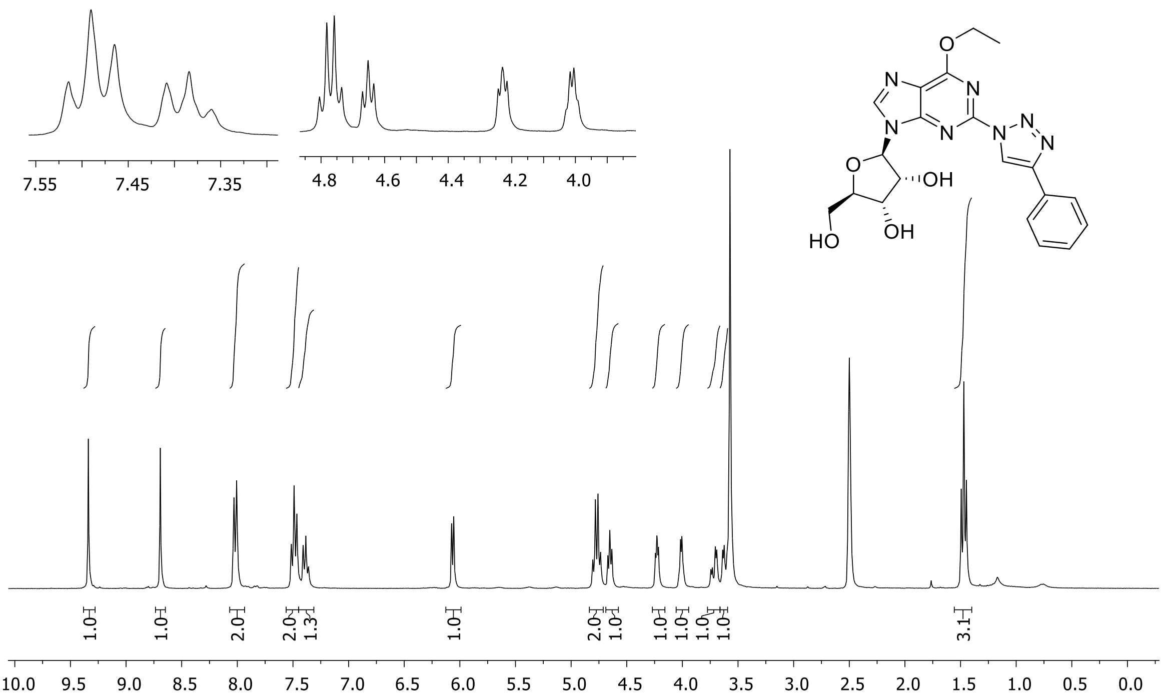

Figure S15: ^{1}H-NMR (300 MHz, DMSO-d_6+D_2O) spectrum.

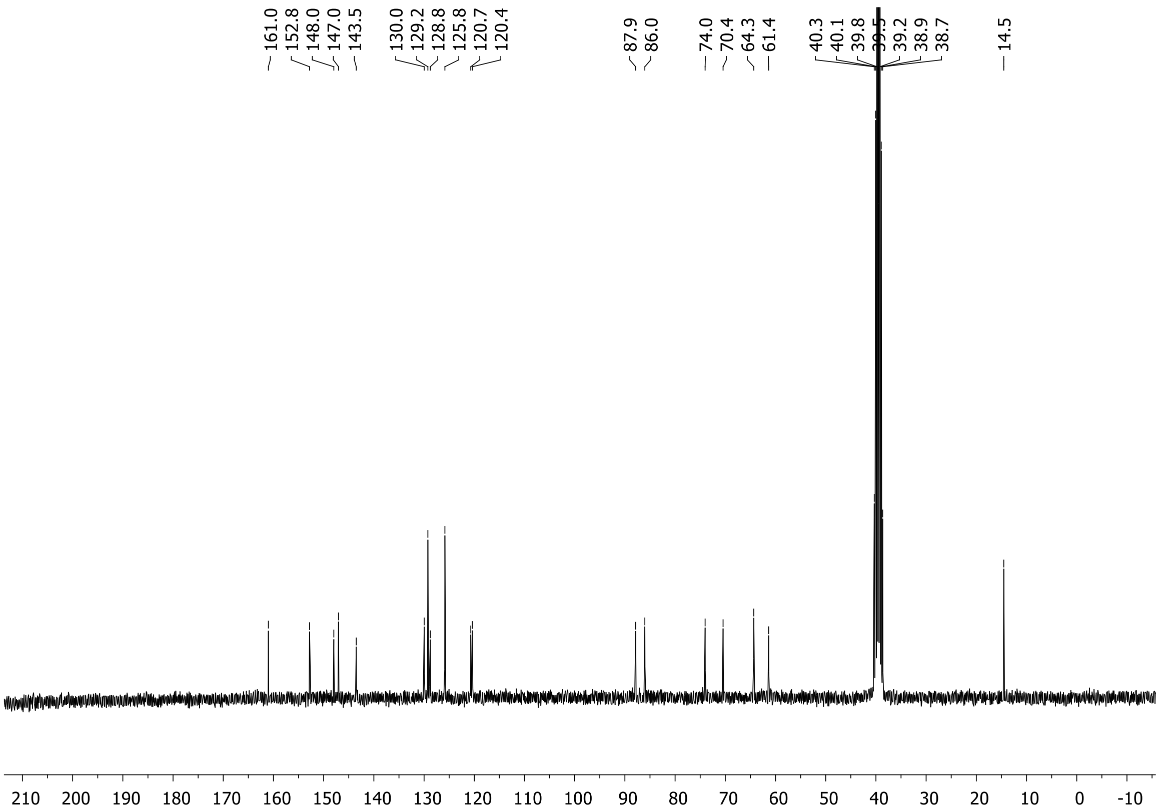

Figure S16: ^{13}C-NMR (75.5 MHz, DMSO-d_6+D_2O) spectrum.

9-β-D-Ribofuranosyl-2-(4-phenyl-1*H*-1,2,3-triazol-1-yl)-6-(prop-1-yl)oxy-9*H*-purine (3i)

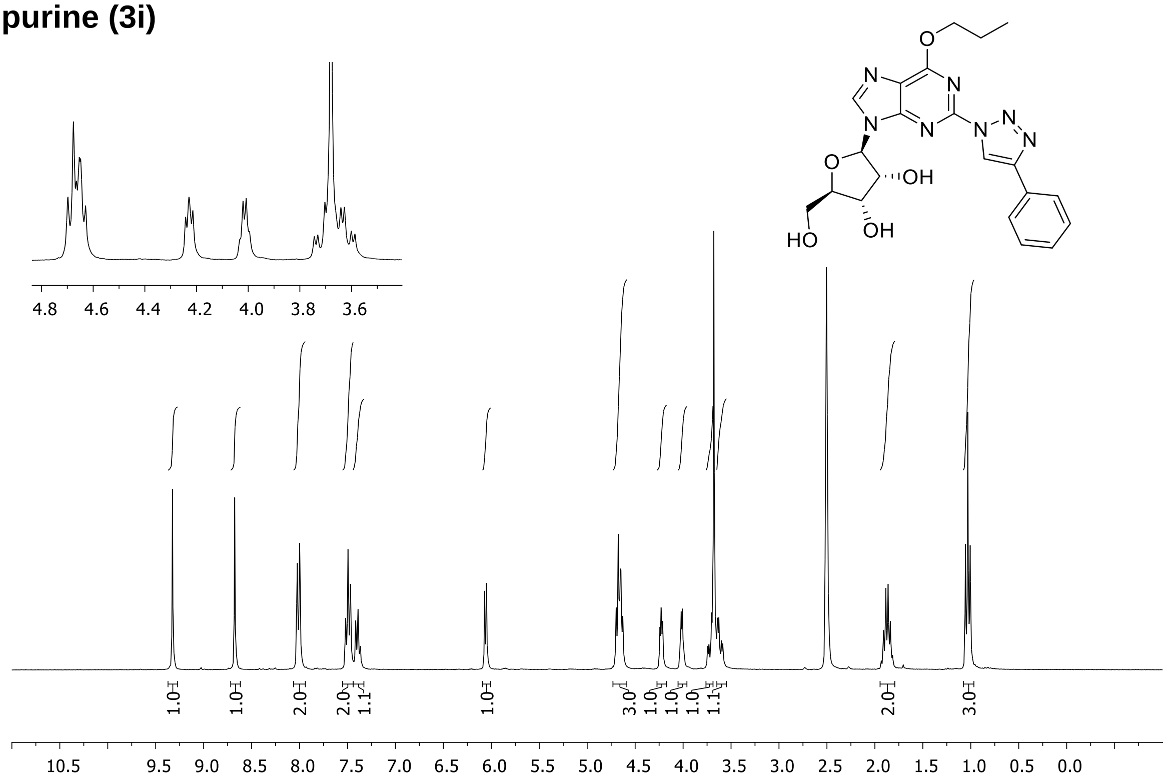

Figure S17: ^{1}H-NMR (300 MHz, DMSO-d_6+D_2O) spectrum.

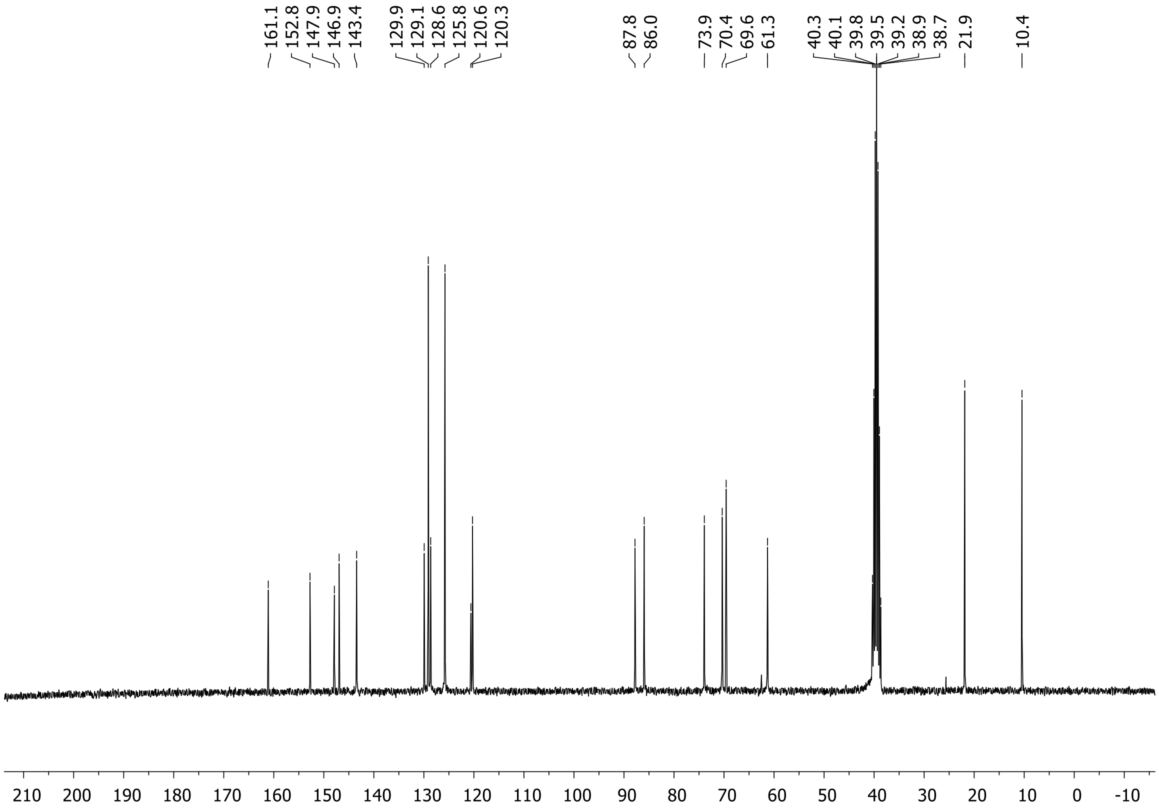

Figure S18: ^{13}C-NMR (75.5 MHz, DMSO-d_6+D_2O) spectrum.

9-(2',3',5'-Tri-*O*-acetyl-*β*-D-ribofuranosyl)-2-(4-phenyl-1*H*-1,2,3-triazol-1-yl)-6-(2''',3'''-isopropylideneuridin-5'''-yl)oxy-9*H*-purine (3j)

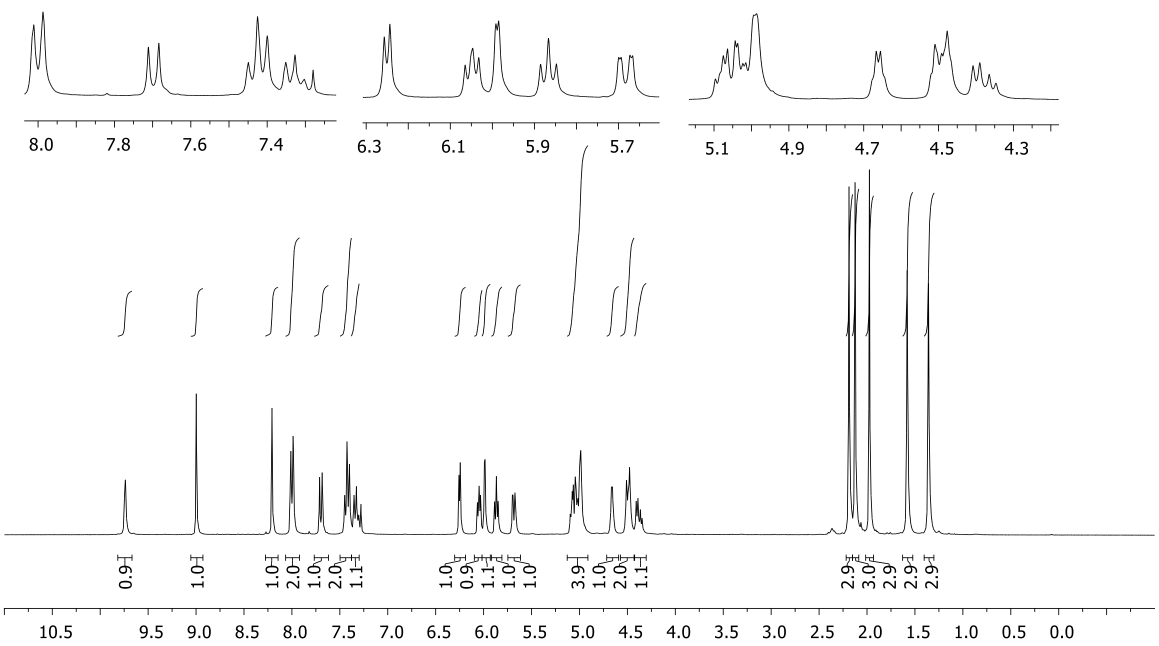

Figure S19: ^{1}H-NMR (300 MHz, $CDCl_3$) spectrum.

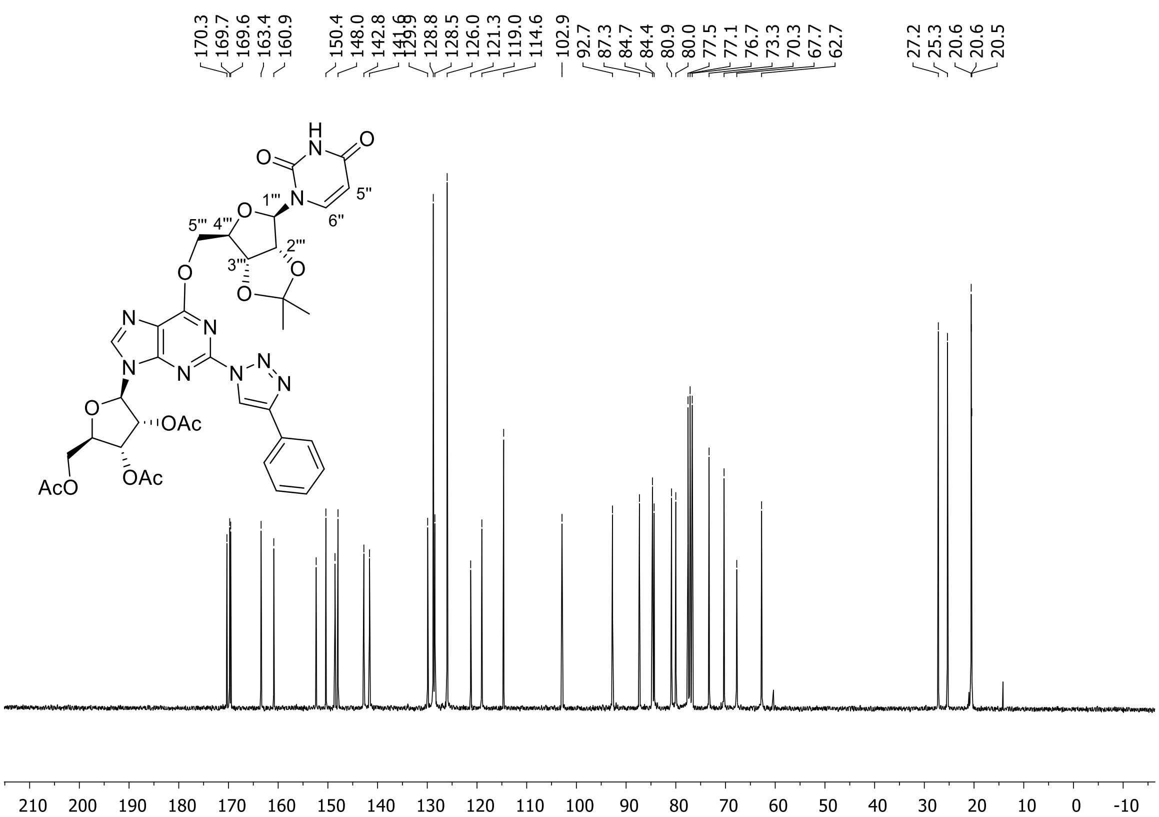

Figure S20: ^{13}C-NMR (75.5 MHz, $CDCl_3$) spectrum.

9-(2',3',5'-Tri-*O*-acetyl-*β*-D-ribofuranosyl)-2-(4-methoxycarbonyl-1*H*-1,2,3-triazol-1-yl)- 6-oxo-1,6-dihydro-9*H*-purine (4a)

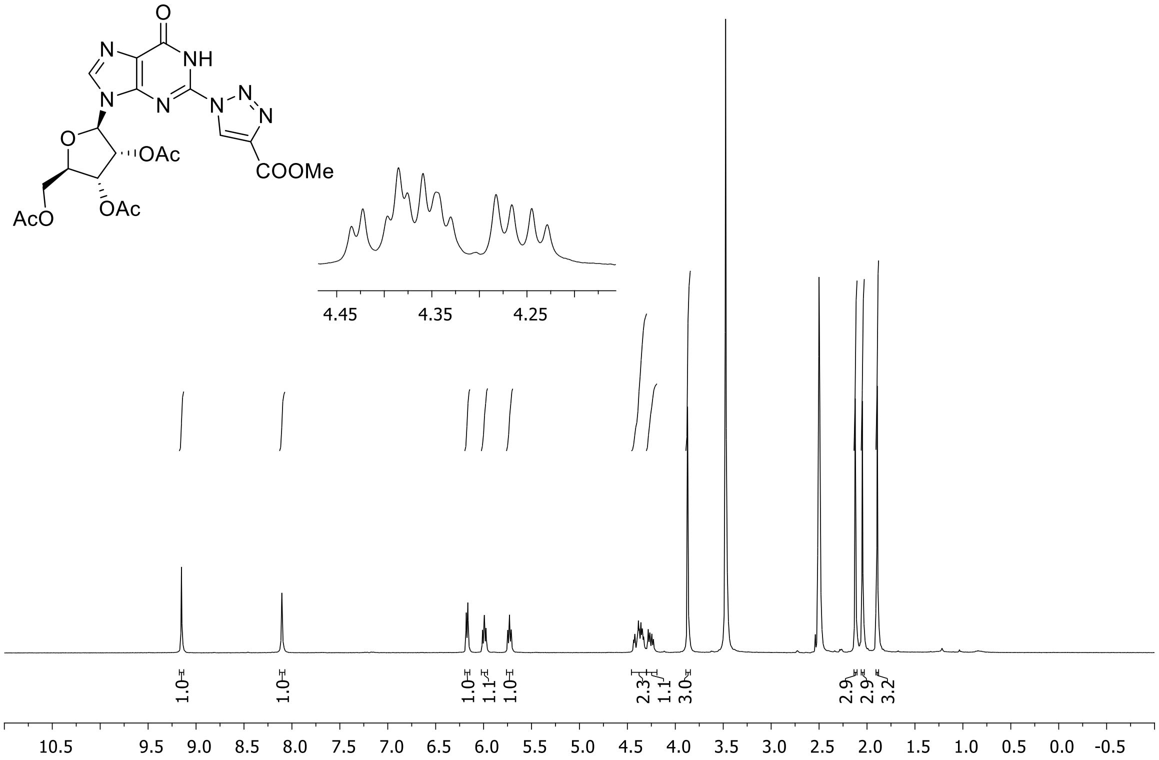

Figure S21: ^{1}H-NMR (300 MHz, DMSO-d$_{6}$+D$_{2}$O) spectrum.

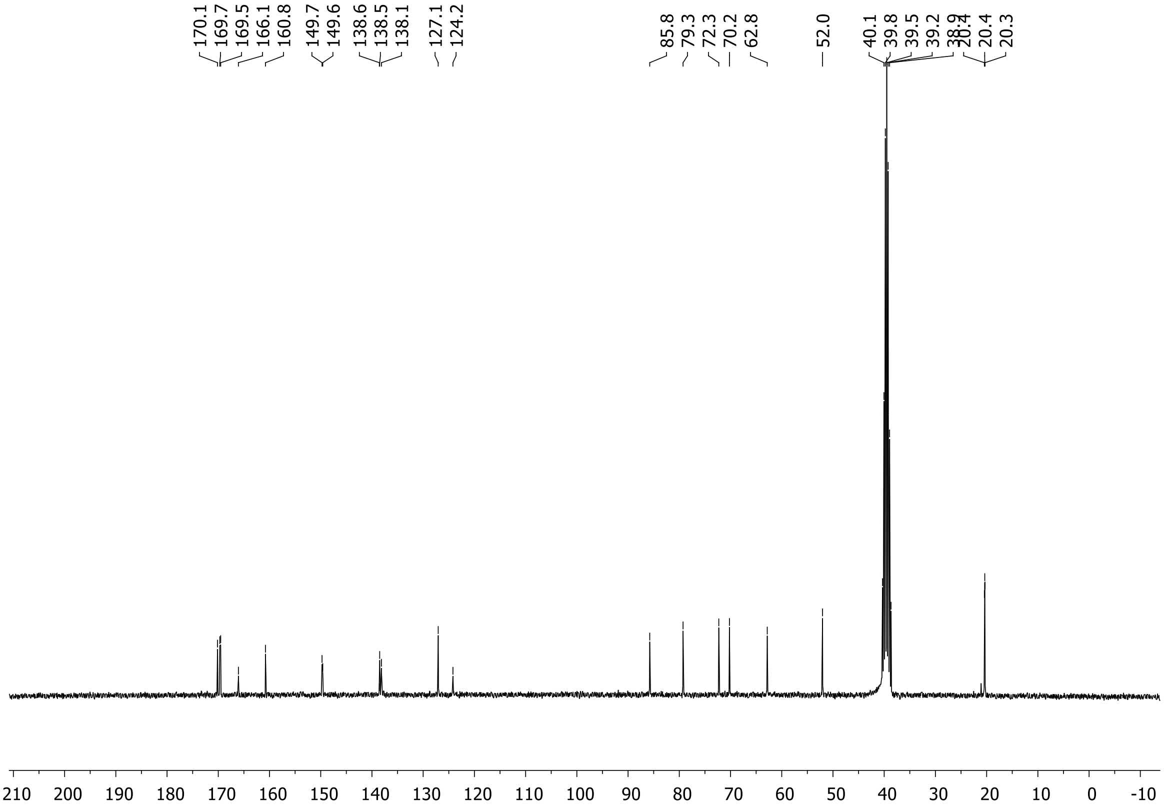

Figure S22: ^{13}C-NMR (75.5 MHz, DMSO-d$_{6}$+D$_{2}$O) spectrum.

9-Heptyl-2-(4-phenyl-1*H*-1,2,3-triazol-1-yl)-1,9-dihydro-6*H*-purin-6-one (4b)

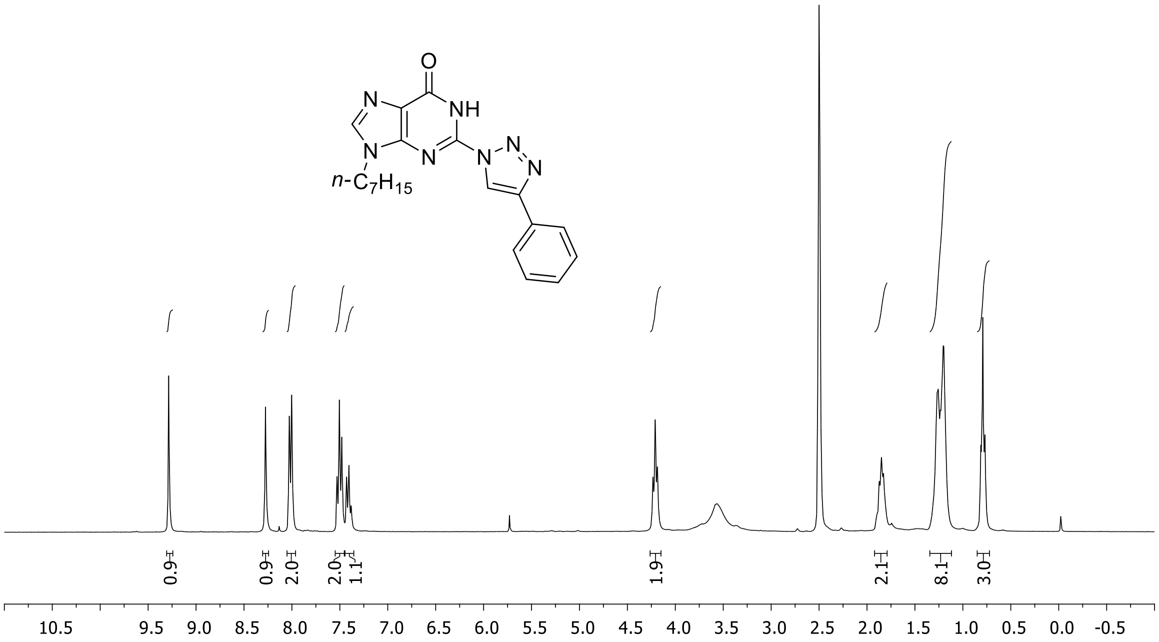

Figure S23: ^{1}H-NMR (300 MHz, DMSO-d$_6$+D$_2$O) spectrum.

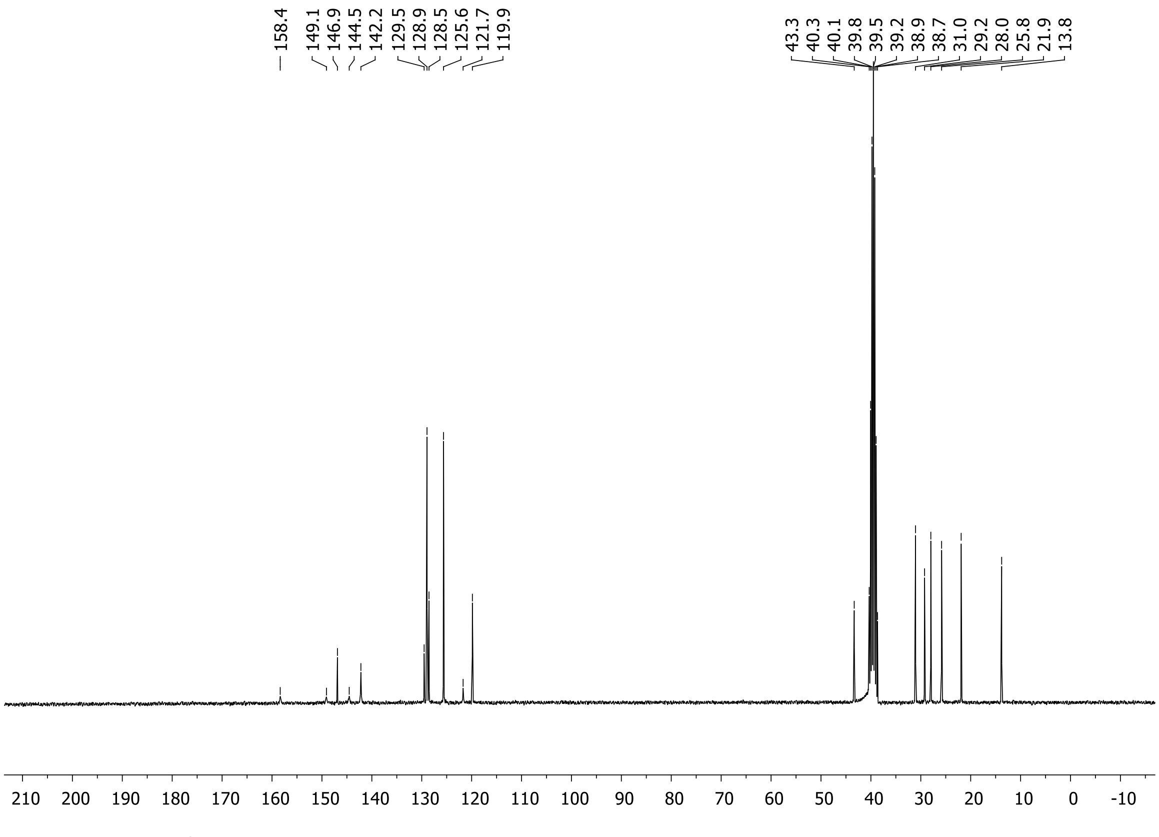

Figure S24: ^{13}C-NMR (75.5 MHz, DMSO-d$_6$+D$_2$O) spectrum.

2-(9-Heptyl-2-(4-phenyl-1*H*-1,2,3-triazol-1-yl)-1,9-dihydro-6*H*-purin-6-ylidene)malononitrile (5a)

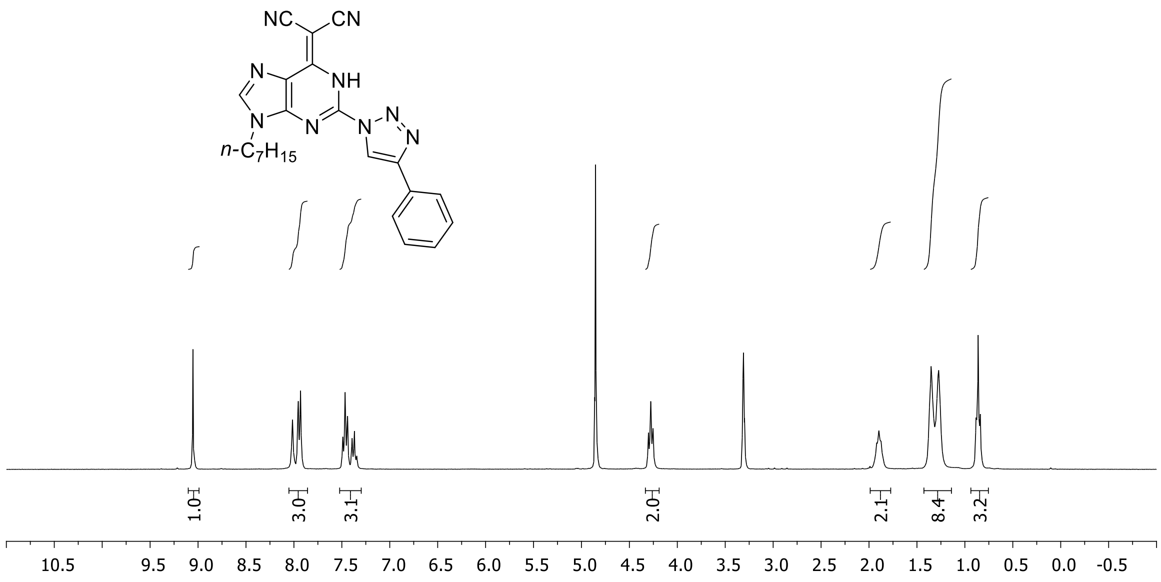

Figure S25: ^{1}H-NMR (300 MHz, CD_3OD +D_2O) spectrum.

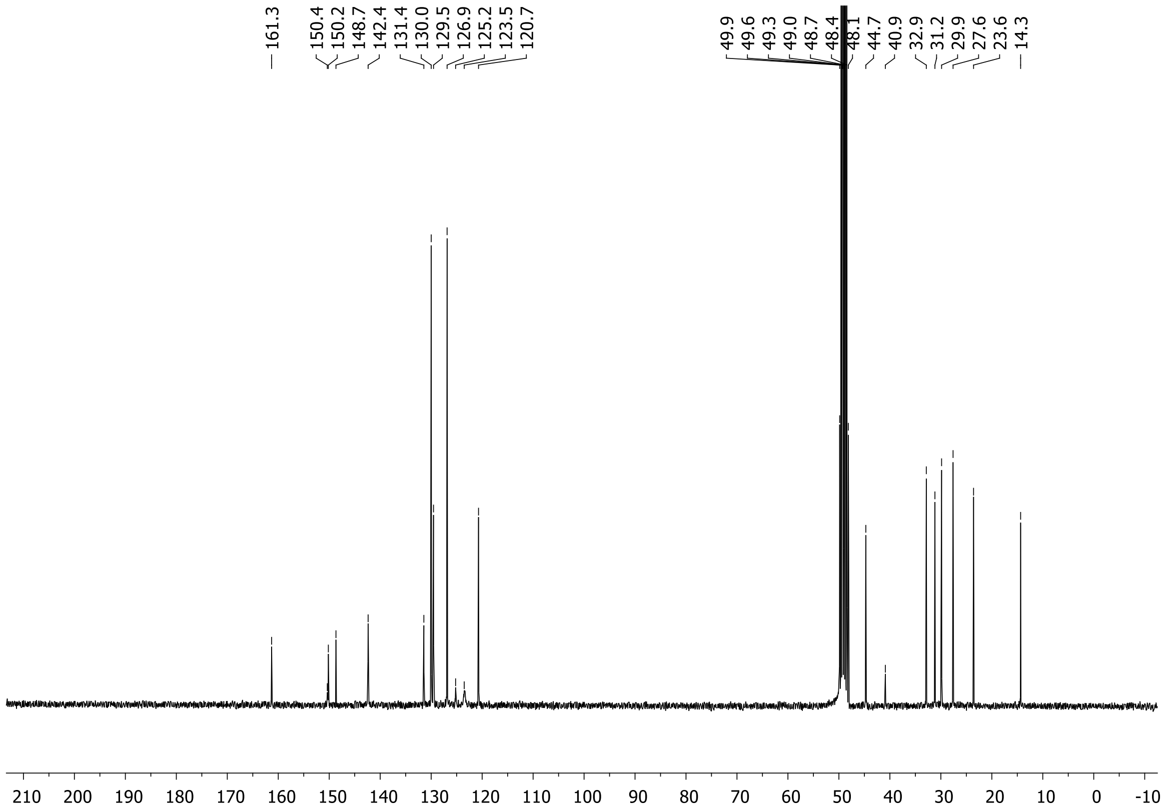

Figure S26: ^{13}C-NMR (75.5 MHz, CD_3OD) spectrum.

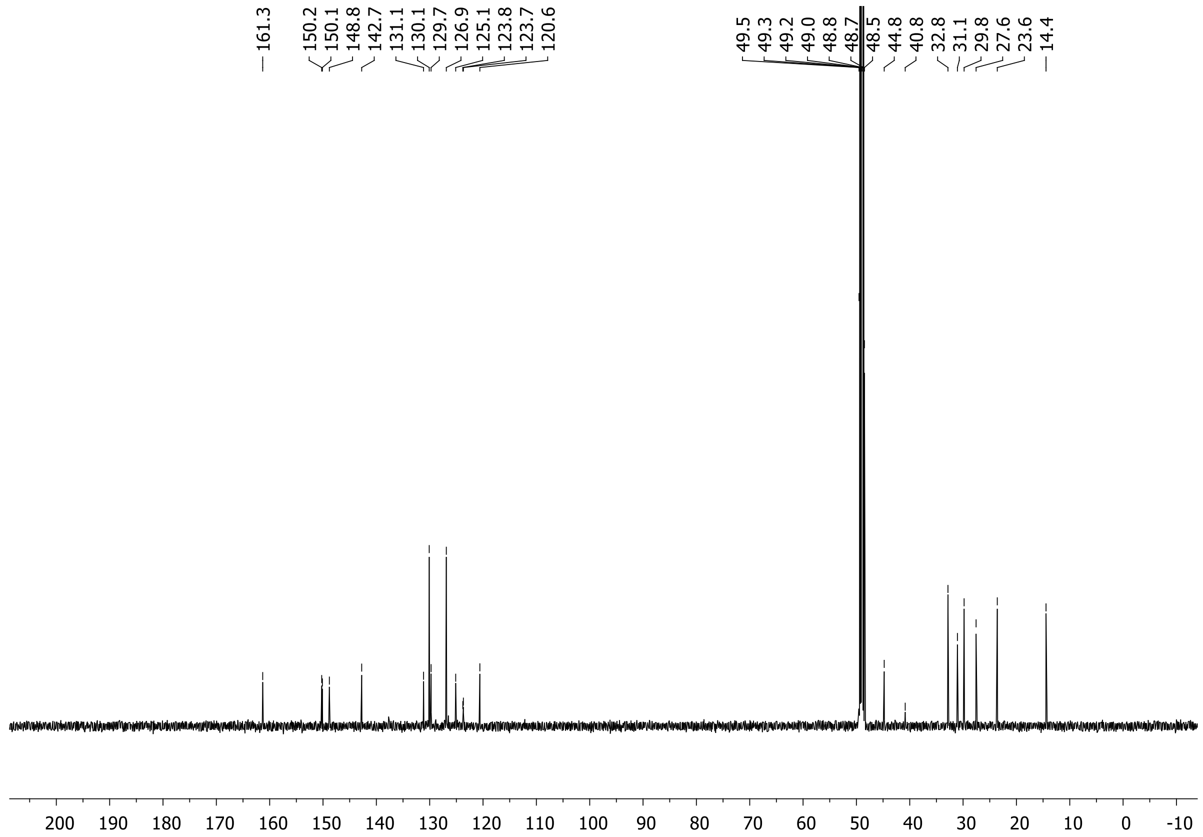

Figure S27: ^{13}C-NMR (125.7 MHz, CD_3OD + NaOD in D_2O (40 w)) spectrum.

2-(9-Heptyl-2-(4-phenyl-1*H*-1,2,3-triazol-1-yl)-9*H*-purin-6-yl)-3-hydroxy-5,5-dimethylcyclohex-2-en-1-one (5b)

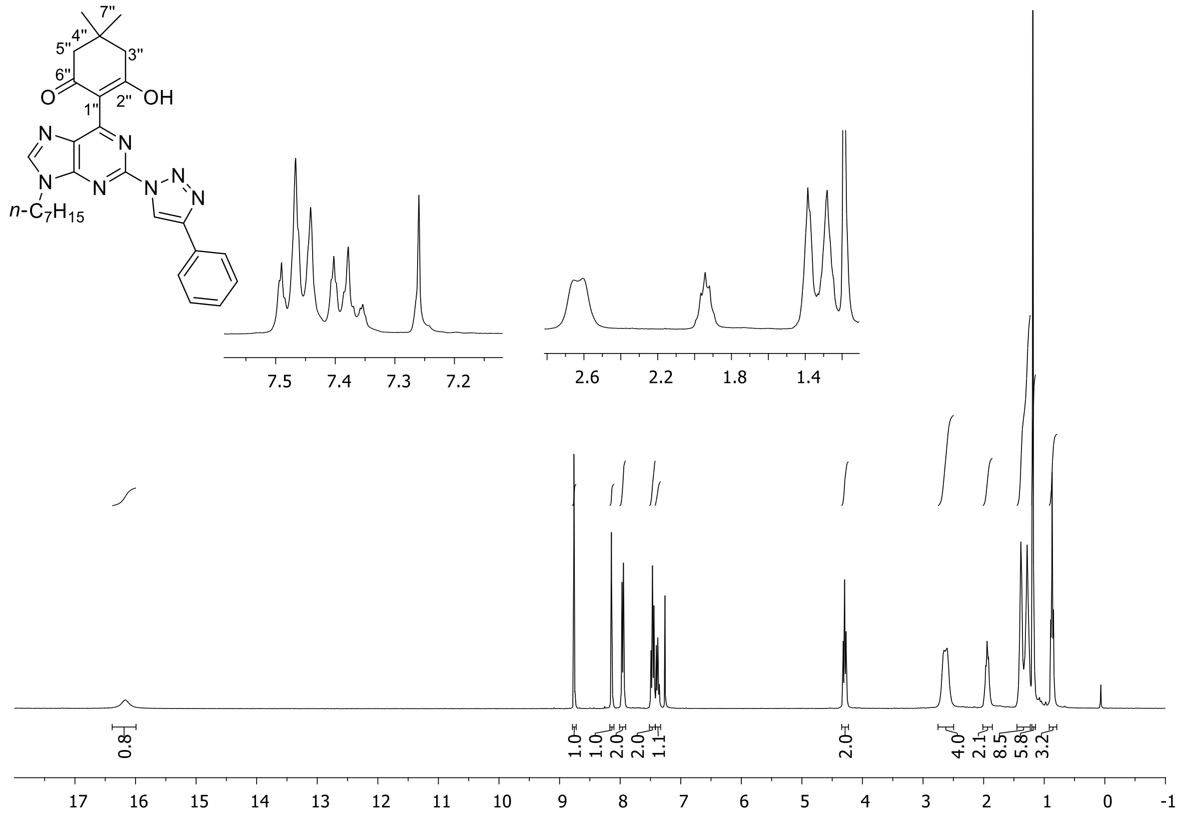

Figure S28: ^{1}H-NMR (300 MHz, $CDCl_3$) spectrum.

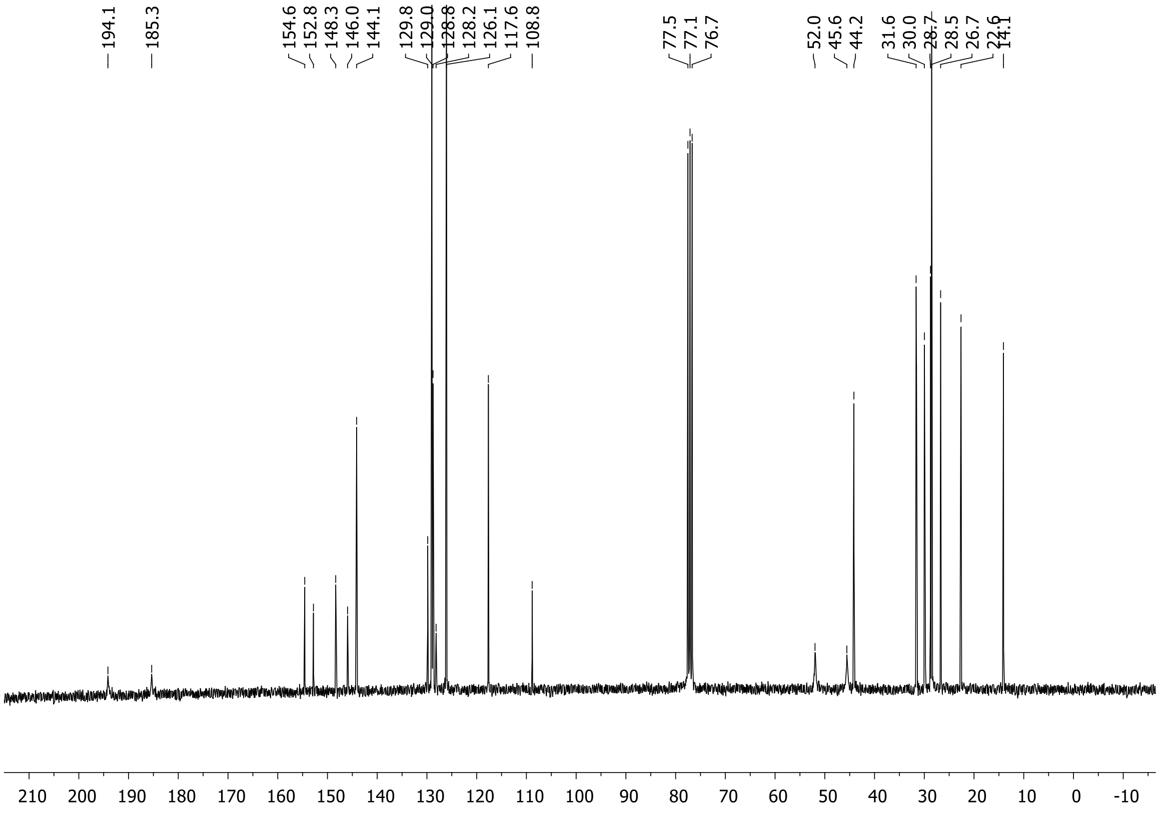

Figure S29: ^{13}C-NMR (75.5 MHz, $CDCl_3$) spectrum.

Ethyl 2-cyano-2-(9-heptyl-2-(4-phenyl-1H-1,2,3-triazol-1-yl)-1,9-dihydro-6*H*-purin-6-ylidene)acetate (5c)

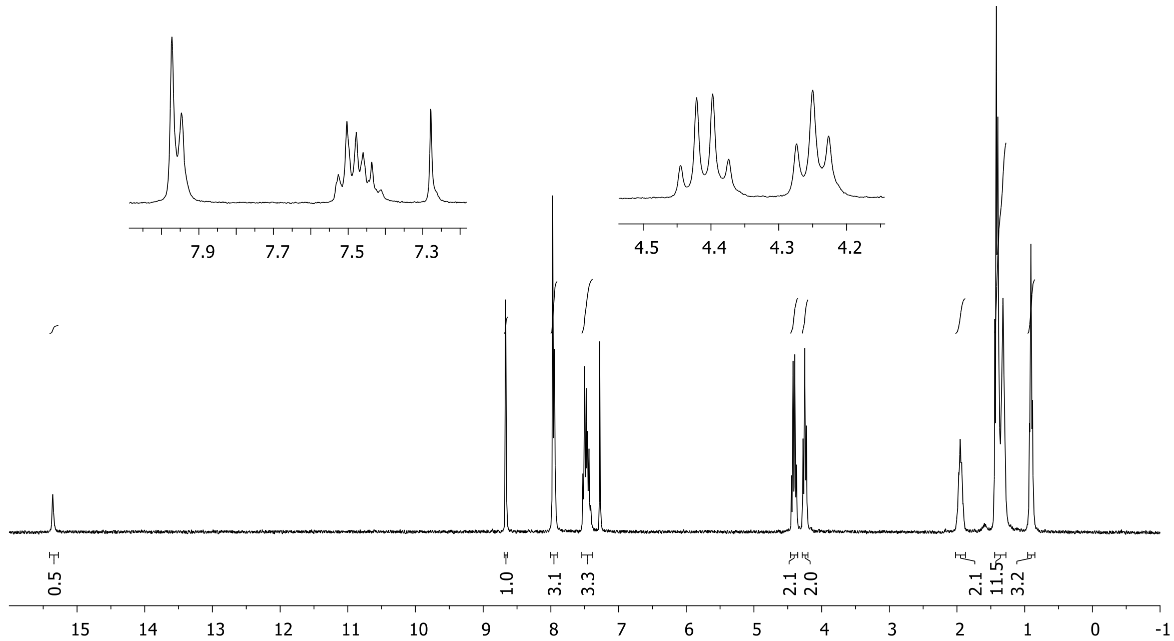

Figure S30: ^{1}H-NMR (300 MHz, $CDCl_3$, 50 ^{o}C) spectrum.

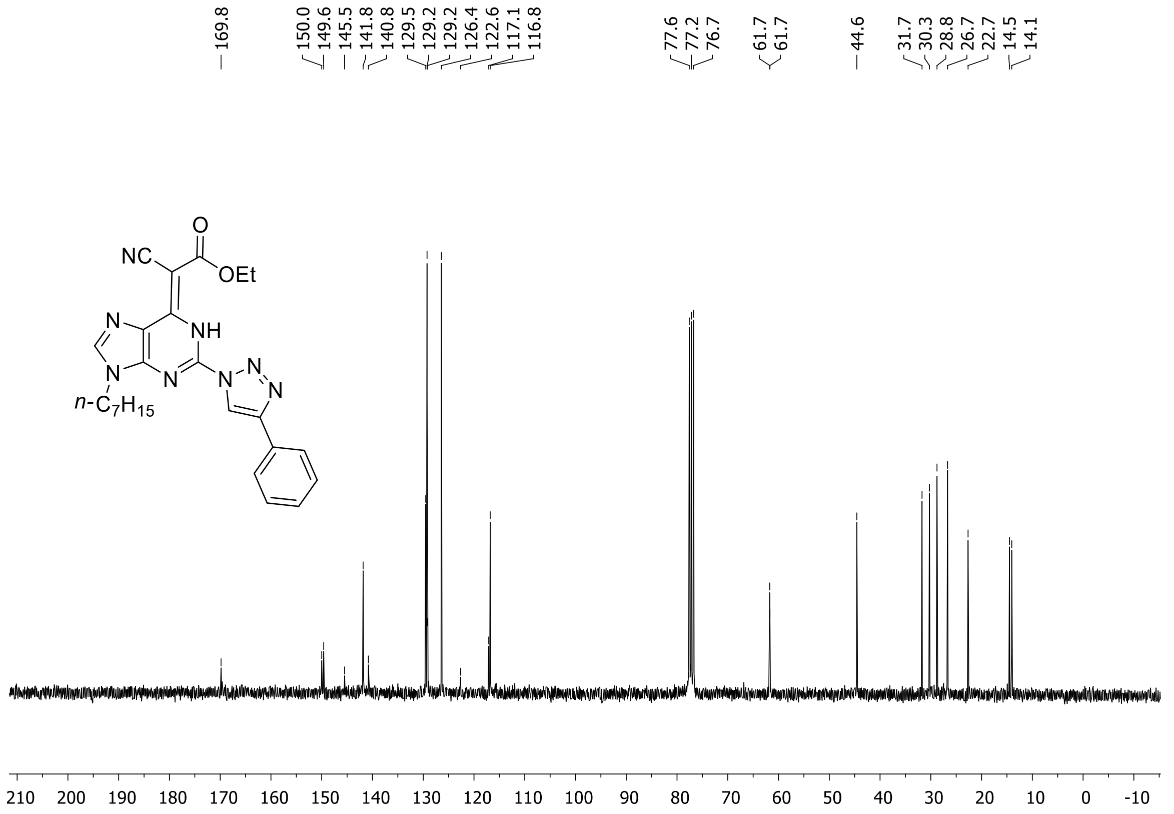

Figure S31: ^{13}C-NMR (75.5 MHz, $CDCl_3$, 50 ^{o}C) spectrum.

Diethyl 2-(9-heptyl-2-(4-phenyl-1*H*-1,2,3-triazol-1-yl)-9*H*-purin-6-yl)malonate (5d)

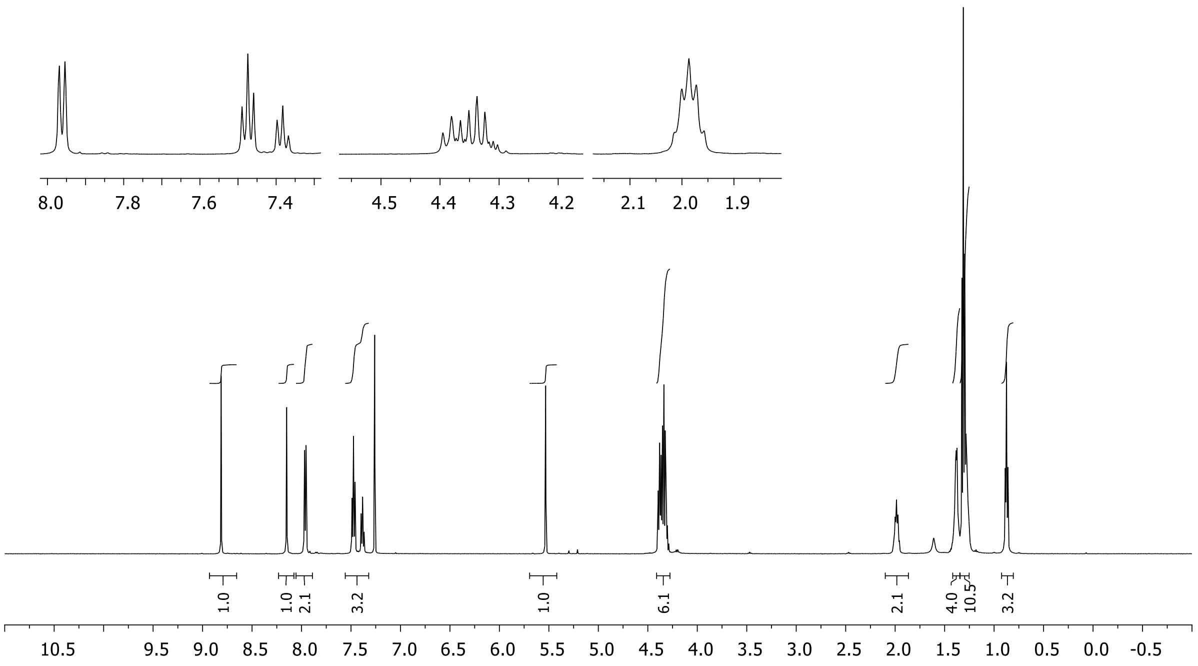

Figure S32: ^{1}H-NMR (500 MHz, $CDCl_3$) spectrum.

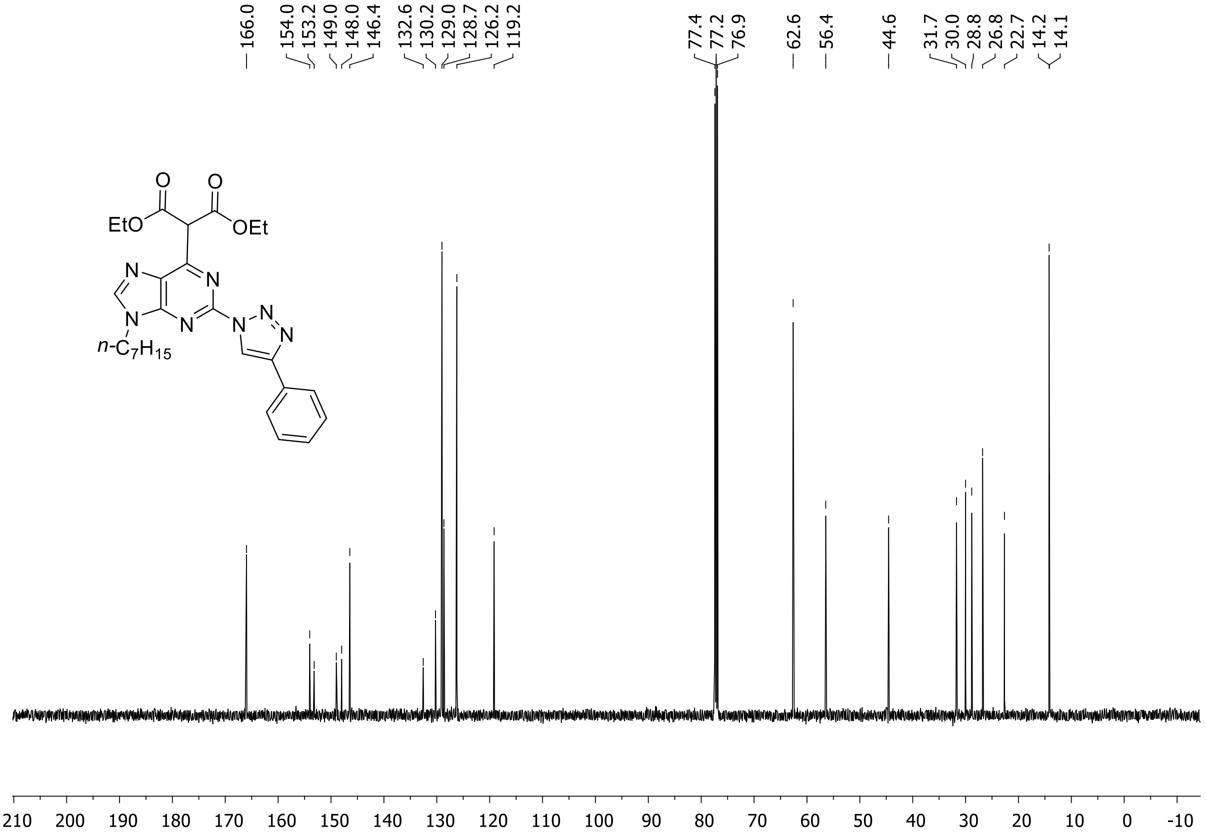

Figure S33: ^{13}C-NMR (125.7 MHz, $CDCl_3$) spectrum.

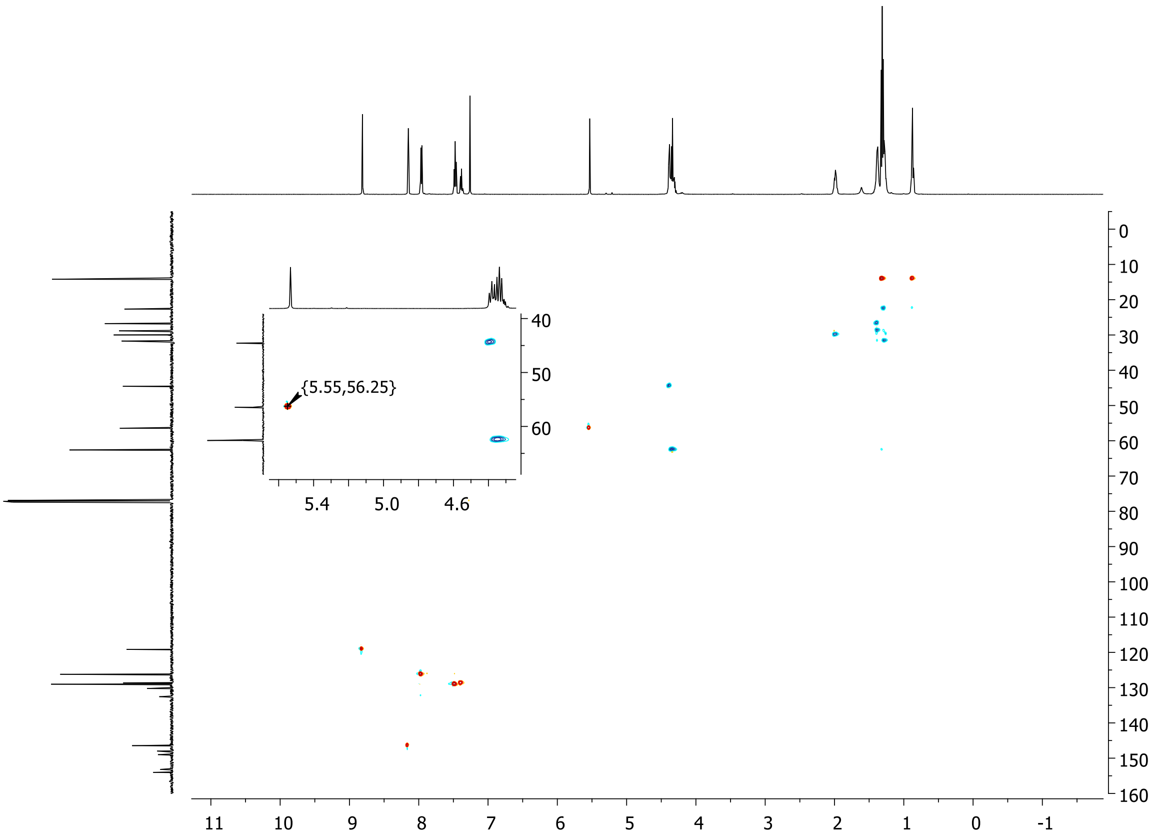

Figure S34: ^{1}H-^{13}C HSQC spectrum of compound **5d**.

References:

1. Kovaļovs, A.; Novosjolova, I.; Bizdēna, Ē.; Bižāne, I.; Skardziute, L.; Kazlauskas, K.; Jursenas, S.; Turks, M. *Tetrahedron Lett.* **2013**, *54*, 850–853. doi:10.1016/j.tetlet.2012.11.095
2. Novosjolova, I.; Bizdēna, Ē.; Turks, M. *Tetrahedron Lett.* **2013**, *54*, 6557–6561. doi:10.1016/j.tetlet.2013.09.095
3. Šišuļins, A.; Bucevičius, J.; Tseng, Y.-T.; Novosjolova, I.; Traskovskis, K.; Bizdēna, Ē.; Chang, H.-T.; Tumkevičius, S.; Turks, M. *Beilstein J. Org. Chem.* **2019**, *15*, 474–489. doi:10.3762/bjoc.15.41
